# Supplementary material for: Treatment burden in survivors of prostate and colorectal cancers: a qualitative interview study
Source: BMJ Open. 2023 Mar 3;13(3):e068997. doi: 10.1136/bmjopen-2022-068997 (PMC9990667; doi:10.1136/bmjopen-2022-068997)
Supplement: Supplementary data [file bmjopen-2022-068997supp001.pdf]

UNIVERSITY OF ABERDEEN

TOPIC GUIDE FOR PATIENT AND CAREGIVER INTERVIEWS

The Improving Cancer Aftercare Study (ICAS). Using patient and caregiver experiences to improve health services for people after prostate and colorectal cancer.

EXPLANATION

Interviews will be directed to some extent by the participants. This document sets out topics that the interviewer will aim to cover. It will be used flexibly.

INTRODUCTIONS

- Introductions and reminder that interview can be paused or stopped at any time. Audio-recording device activated.
- From previous research, we are aware that looking after yourself after cancer can involve quite a bit of work. We'd like to explore that with you.

CONTEXT

- Perhaps I can start by asking you about how your health is generally?
  - Potential prompts – comorbidities, taking multiple medications, function, employment, activity levels
- How confident do you feel about managing your health conditions? (For example, remembering to take medicines, getting help and advice when needed) Participant will be prompted to explore any difficulties

PLANNING MANAGEMENT: INITIAL DIAGNOSTIC AND TREATMENT PERIOD

- Thinking back to when you were initially diagnosed with prostate/colorectal cancer, can you tell me the story of that?

- What was important to you about the healthcare that you received then? (Prompts might include ease of access, continuity, regular appointments, information and planning, communication with health service)

- What went well?

- Were there any aspects of your care that could have been improved?

- Did you get the information that you needed? (prompts might include what kind of information was important, who was approached/available for information e.g. charities, professionals, social network)

- Can you tell me about any ways that your life changed after your diagnosis of prostate/colorectal cancer? (Prompts – more appointments, travel, new medicines, dietary changes, smoking cessation, exercise, psychological adjustment)

- I have heard it said that cancer management can be a "full time job". Do you have any thoughts about that?

Adam R, *et al. BMJ Open* 2023; 13:e068997. doi: 10.1136/bmjopen-2022-068997

INTERACTING WITH OTHERS

- Did anything/or anyone help you manage?
- How often were you attending hospital? (After diagnosis, during treatment, now)
- How easy or difficult is/was it for you to get help from healthcare professionals? (prompts – who was involved, specialist nurse, primary care, nursing care, has this changed over time/throughout the illness trajectory, physical access and distance to healthcare settings)
- Have family and friends been involved in any way? (Prompts might include transport, emotional support, information)
- Do you get regular tests or investigations?
- How easy/difficult do you find it to get results of your tests?

ENACTING CURRENT MANAGEMENT STRATEGIES AND GETTING FEEDBACK

Does cancer affect any aspects of a typical day or week at the moment?

  - Prompts – symptoms, self-monitoring, psychological adjustment, taking medications, strategies to remember medications

- Have you changed any of your routines or aspects of your lifestyle?
  - Prompts – diet, exercise, smoking, ***employment***, activities, ***social function***, have professionals asked the patient to make any changes?
- How confident have you felt about making these changes?
- What is the plan for your cancer follow-up? (Prompts – schedule of tests and clinic appointments, tasks that are required from patient/caregiver e.g. monitoring symptoms, taking medications, duration of follow-up, understanding of plan
- Have you had any problems scheduling or attending appointments?

SATISFACTION, SUGGESTIONS, AND SUMMING UP

- Overall, how satisfied are you with the care that you have received following your cancer diagnosis?
- Have you any suggestions for how the care could be improved?
- Some healthcare systems allow people to book or change appointments on-line, get reminders about follow-up tests, and access some results. Have you any thoughts about this?

We have covered lots of things (brief summary) – is there anything else that you would like to speak about that we haven't covered? Participant is thanked for their time.

END of INTERVIEW

Patient and carer interview topic guide. Version 1. 25th July 2019. IRAS ID 269788
