# Supplementary figures and images for: Treatment burden in survivors of prostate and colorectal cancers: a qualitative interview study
Source: BMJ Open. 2023 Mar 3;13(3):e068997. doi: 10.1136/bmjopen-2022-068997 (PMC9990667; doi:10.1136/bmjopen-2022-068997)

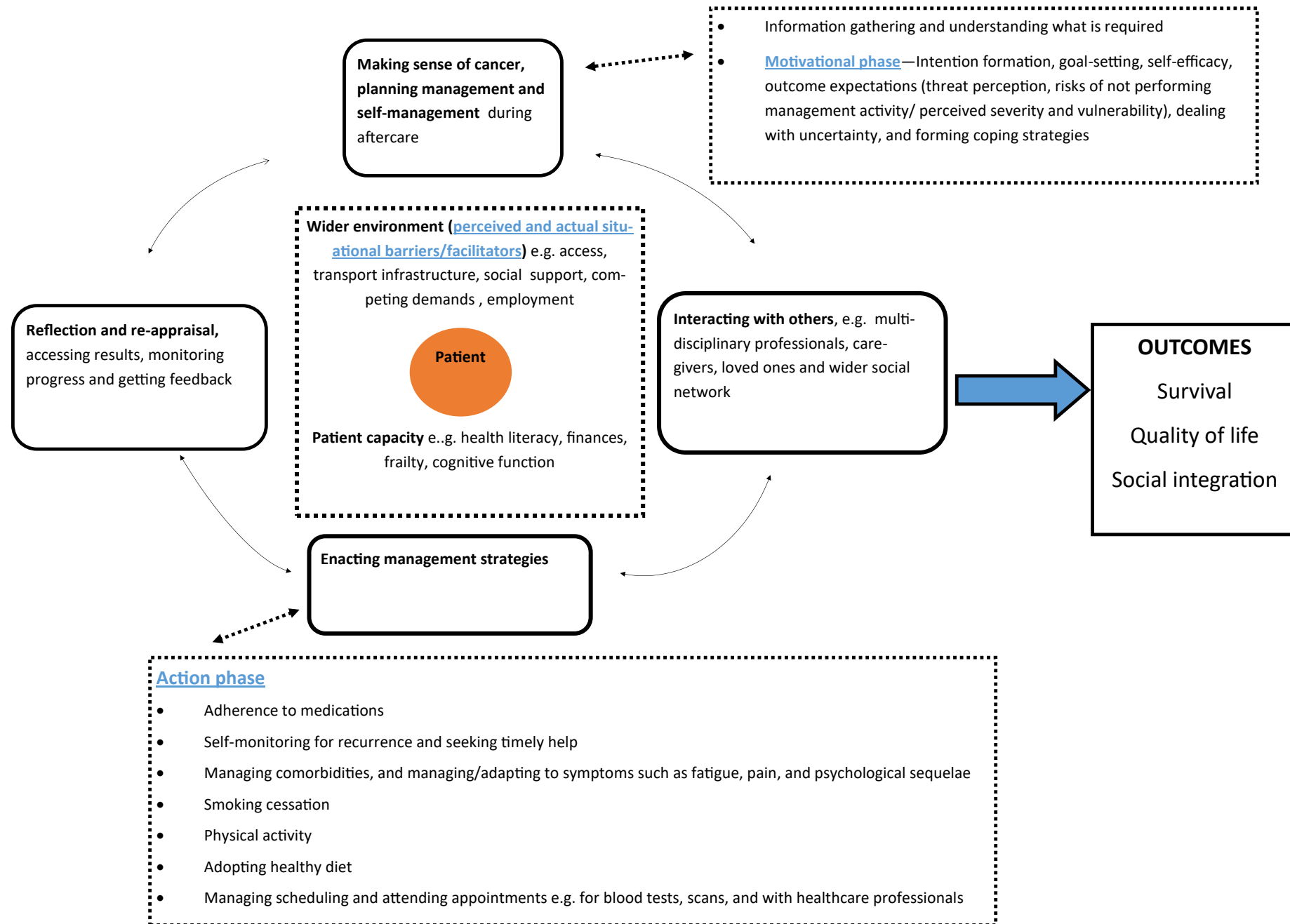

Supplement: Supplementary data [file bmjopen-2022-068997supp003.pdf]
