# Supplementary material for: Treatment burden in survivors of prostate and colorectal cancers: a qualitative interview study
Source: BMJ Open. 2023 Mar 3;13(3):e068997. doi: 10.1136/bmjopen-2022-068997 (PMC9990667; doi:10.1136/bmjopen-2022-068997)
Supplement: Supplementary data [file bmjopen-2022-068997supp002.pdf]

Supplementary Table 1. Comparisons in demographics between participants and non-participants.

	**Participants (N = 35)** N (%)	**Non-participants invited (N = 128)** N (%)	**Chi-square test of independence or Fisher Exact test *p*-value**
**Sex**			
Male	28 (80%)	105 (82%)	0.98
Female	7 (20%)	23 (18%)	
**Age**			
30-39	1 (2.9%)	0 (0%)	
40-49	0 (0%)	1 (0.8%)	
50-59	3 (8.6%)	16 (12.5%)	0.46
60-69	14 (40%)	40 (31.3%)	
70-79	14 (40%)	54 (42.2%)	
80+	3 (8.6%)	17 (13.3%)	
**Deprivation Index**			
1-2 (Highest deprivation)	2 (5.7%)	20 (15.6%)	
3-4	5 (14.3%)	34 (26.6%)	
5-6	7 (20%)	22 (17.2%)	0.06
7-8	11 (31.4%)	20 (15.6%)	
9-10 (Lowest deprivation)	9 (25.7%)	32 (25%)	
Missing	1 (2.9%)	0 (0%)	
**Urban Rural Classification**			
1 (Large urban)	17 (48.6%)	85 (66.4%)	
2 (Other urban)	1 (2.9%)	5 (3.9%)	
3 (Accessible small town)	2 (5.7%)	7 (5.5%)	0.09
4 (Remote small town)	0 (0%)	0 (0%)	
5 (Accessible rural)	10 (28.6%)	16 (12.5%)	
6 (Remote rural)	4 (11.4%)	15 (11.7%)	
Missing	1 (2.9%)	0 (0%)	
**Cancer type**			
Prostate	22 (62.9%)	80 (62.5%)	
Colorectal	13 (37.1%)	45 (35.2%)	1
Not specified	0	3 (2.3%)	
