# Supplementary material for: Treatment burden in survivors of prostate and colorectal cancers: a qualitative interview study
Source: BMJ Open. 2023 Mar 3;13(3):e068997. doi: 10.1136/bmjopen-2022-068997 (PMC9990667; doi:10.1136/bmjopen-2022-068997)
Supplement: Author's manuscript [file bmjopen-2022-068997.draft_revisions.pdf]

BMJ Open

BMJ Open is committed to open peer review. As part of this commitment we make the peer review history of every article we publish publicly available.

When an article is published we post the peer reviewers' comments and the authors' responses online. We also post the versions of the paper that were used during peer review. These are the versions that the peer review comments apply to.

The versions of the paper that follow are the versions that were submitted during the peer review process. They are not the versions of record or the final published versions. They should not be cited or distributed as the published version of this manuscript.

BMJ Open is an open access journal and the full, final, typeset and author-corrected version of record of the manuscript is available on our site with no access controls, subscription charges or pay-per-view fees (http://bmjopen.bmj.com).

If you have any questions on BMJ Open's open peer review process please email info.bmjopen@bmj.com

BMJ Open

Treatment burden in survivors of prostate and colorectal cancer: a qualitative interview study

Journal:	*BMJ Open*
Manuscript ID	bmjopen-2022-068997
Article Type:	Original research
Date Submitted by the Author:	14-Oct-2022

Complete List of Authors:	Adam, Rosalind; University of Aberdeen, Academic Primary Care, Institute of Applied Health Sciences<br>Duncan, Lisa; University of Aberdeen, Academic Primary Care, Institute of Applied Health Sciences<br>Maclennan, Sara; University of Aberdeen, Academic Urology Unit<br>Locock, Louise; University of Aberdeen, Health Services Research Unit

Keywords:	QUALITATIVE RESEARCH, Adult oncology < ONCOLOGY, Quality in health care < HEALTH SERVICES ADMINISTRATION & MANAGEMENT, Gastrointestinal tumours < ONCOLOGY, Urological tumours < ONCOLOGY

SCHOLARONE™
Manuscripts

I, the Submitting Author has the right to grant and does grant on behalf of all authors of the Work (as defined in the below author licence), an exclusive licence and/or a non-exclusive licence for contributions from authors who are: i) UK Crown employees; ii) where BMJ has agreed a CC-BY licence shall apply, and/or iii) in accordance with the terms applicable for US Federal Government officers or employees acting as part of their official duties; on a worldwide, perpetual, irrevocable, royalty-free basis to BMJ Publishing Group Ltd ("BMJ") its licensees and where the relevant Journal is co-owned by BMJ to the co-owners of the Journal, to publish the Work in this journal and any other BMJ products and to exploit all rights, as set out in our licence.

The Submitting Author accepts and understands that any supply made under these terms is made by BMJ to the Submitting Author unless you are acting as an employee on behalf of your employer or a postgraduate student of an affiliated institution which is paying any applicable article publishing charge ("APC") for Open Access articles. Where the Submitting Author wishes to make the Work available on an Open Access basis (and intends to pay the relevant APC), the terms of reuse of such Open Access shall be governed by a Creative Commons licence – details of these licences and which Creative Commons licence will apply to this Work are set out in our licence referred to above.

Other than as permitted in any relevant BMJ Author's Self Archiving Policies, I confirm this Work has not been accepted for publication elsewhere, is not being considered for publication elsewhere and does not duplicate material already published. I confirm all authors consent to publication of this Work and authorise the granting of this licence.

For peer review only

Treatment burden in survivors of prostate and colorectal cancer: a qualitative interview study

Dr Rosalind Adam, Academic Primary Care, Institute of Applied Health Sciences, University of Aberdeen. Room 1:020, Polwarth Building, Foresterhill, Aberdeen, AB25 2ZD. rosalindadam@abdn.ac.uk; 01224 437906. (Corresponding Author).

Miss Lisa Duncan, Academic Primary Care, Institute of Applied Health Sciences, University of Aberdeen. Room 1:023, Polwarth Building, Foresterhill, Aberdeen, AB25 2ZD.

Dr Sara MacLennan, Academic Urology Unit, Institute of Applied Health Sciences, Health Sciences Building (2nd Floor), University of Aberdeen, Foresterhill, Aberdeen, AB25 2ZD.

Professor Louise Locock, Health Services Research Unit, Institute of Applied Health Sciences, Health Sciences Building (2nd Floor), University of Aberdeen, Foresterhill, Aberdeen, AB25 2ZD.

Acknowledgements

The authors would like to thank the participants who volunteered their time to give detailed accounts of their experiences. We would also like to thank the National Health Service Research Scotland Primary Care Network, who facilitated patient recruitment via general practices. We thank the general practices who participated.

Funding Statement

This work was funded by a Scottish Government Chief Scientist Office Senior Clinical Academic Fellowship awarded to Rosalind Adam, reference SCAF/18/02.

Competing interests

The authors have no relevant financial or non-financial interests to disclose.

For peer review only

Abstract

Objectives: Treatment burden is the workload of health care and the impact this has on the individual. Treatment burden is associated with poorer patient outcomes in several chronic diseases. Illness burden has been extensively studied in cancer, but little is known about treatment burden, particularly in those who have completed primary treatment for cancer. The aim of this study was to investigate treatment burden in survivors of prostate and colorectal cancer and their caregivers.

Design: Semi-structured interview study. Interviews were analysed using Framework and thematic analysis.

Setting: Participants were recruited via general practices in Northeast Scotland

Participants: Eligible participants were individuals who had been diagnosed with colorectal or prostate cancer without distant metastases within the previous five years and their caregivers. Thirty-five patients and 6 caregivers participated: 22 patients had prostate and 13 had colorectal cancers (6, male, 7 female).

Results

The term "burden" did not resonate with most survivors, who expressed gratitude that time invested in cancer care could translate into improved survival. Cancer management was time consuming, but workload reduced over time. Cancer was usually considered as a discrete episode. Individual-, disease-, and health system- factors protected against or increased treatment burden. Some factors, such as health service configuration were potentially modifiable. Multimorbidity contributed most to treatment burden and could influence treatment decisions and engagement with follow up.

Conclusions

Intensive cancer treatment and follow-up regimes do not necessarily lead to perceived burden. A cancer diagnosis serves as a strong motivator to engage in health management, but

For peer review only

a careful balance exists between empowerment and burden. Treatment burden could lead to poorer cancer outcomes by influencing engagement with and decisions about care. Clinicians should ask about treatment burden and its impact, particularly in those with multimorbidity.

Trial Registration: NCT04163068; clinicaltrials.gov

Strengths and Limitations

- In-depth interviews with 41 individuals, generating rich qualitative data
- Participant feedback on the results and key messages
- The study was conducted in a single geographical area
- Participants from areas of higher social deprivation were relatively under-represented

Word count = 3733 including quotations (3013 minus participant quotations)

Keywords – Qualitative research, adult oncology, quality in health care, gastrointestinal tumours, urological tumours

For peer review only

Introduction

Modern cancer survivorship care places physical, financial, psychosocial, and practical demands on individuals and their families [1–4]. There is a move away from hospital and clinician-led cancer care towards supported self-monitoring and self-management by patients and their families in the community [5, 6].

Treatment burden is the workload of healthcare and the impact that this work has on the individual [7]. Treatment burden is of considerable and increasing importance [8]due to ageing populations, a rising prevalence of multimorbidity [9, 10], and increased pressure on healthcare systems. Health care workload is increasingly being transferred from clinicians and healthcare systems towards patients and their families[11]. It is held that individuals who become overburdened by the workload of health care disengage from self-management activities, leading to poorer outcomes [12–14].

Treatment burden is likely to be important in cancer survivors[1]. Questionnaire studies have detected high levels of treatment burden after cancer, particularly in those with lower health literacy [15, 16], multiple comorbidities [15–17], and lower social support [15, 17]. One qualitative interview study investigated treatment burden in individuals who were undergoing or had recently completed treatment for lung cancer [18]. Patients had restructured their lives to accommodate treatment-associated workload. This was accepted by patients as a necessity due to the severe and life-threatening nature of lung cancer [18]. It is unclear how treatment burden might be perceived by survivors of cancer in whom the prognosis is more favourable than lung cancer.

Prostate and colorectal cancers are the third and fourth most common invasive cancers worldwide [19]. Over 90% of men with prostate cancer are diagnosed with local or regional

[1] The term “survivor” is used here to describe individuals living with and beyond cancer. It is a term that is widely used in research, guidelines, and charities but which can divide opinion in some individuals with cancer.

For peer review only

disease, for which the disease specific 5-year survival approaches 100% [20]. Improvements in the detection of colorectal cancer mean that 76% of colorectal cancers are diagnosed at local or regional stages [3]. Prostate and colorectal cancers encompass a wide range of treatment modalities and follow-up activities. They are ideal exemplar cancers through which to study the burden of survivorship care and whether treatment burden could influence outcomes in cancer survivors.

The aim of this study was to investigate perceptions of treatment burden in individuals who had completed treatment for colorectal cancer or prostate cancer and their caregivers and the impact of treatment burden on these individuals.

Methods

Setting and design

A qualitative semi-structured interview study was conducted in NHS Grampian in Northeast Scotland. Cancer care is centred around a university teaching hospital with academic links, and care pathways that are integrated with local cancer charities.

Participants and recruitment

Eligible participants were adults with a history of localised or locally advanced prostate or colorectal cancer, diagnosed within the past five years. A five-year cut off was chosen so that individuals were reflecting on recent experiences of cancer treatment and follow-up, and because many individuals with low-risk disease are discharged from follow up after five years. Patients could also nominate a caregiver to participate in the interview if they wished. Participants were included if they had received any cancer treatment/management, including, and not limited to active surveillance, surgery, radiotherapy, or chemotherapy. Individuals who were undergoing or on waiting lists for chemotherapy, radiotherapy, or surgery were excluded because their experiences of treatment burden were likely to be different from individuals who were in the follow-up stages after active cancer treatment. Those with distant

For peer review only

metastases were excluded because treatment and follow-up for these individuals would have different aims and formats of delivery.

NHS Research Scotland Primary Care (NRS Primary Care) assisted with recruitment. NRS Primary Care recruited general practices and searched electronic medical records using Read codes for prostate and colorectal cancer. General practices sent invitation packs to eligible patients, and those interested in participating responded directly to the research team.

Data collection and management

An interview topic guide was designed to ensure comprehensive coverage of the topic. A conceptual model (Supplementary Figure 1) was produced, drawing on existing literature on treatment burden in stroke [21], Normalisation Process Theory [22], and the Health Action Process Approach [23]. The model was used to inform the topic guide, which included questions about the initial diagnosis and treatment period, current self-management strategies, social support, and interactions with others. Participants were asked to reflect on the work of cancer management and any effects that managing their health had on them. The guide was used flexibly, allowing the interview to be directed by participant responses.

The study commenced in January 2020 and was put on hold in March 2020 during the Covid-19 pandemic, restarting in May 2021. Interviews conducted prior to March 2020 were conducted face to face or by telephone, according to participant preference. All interviews undertaken after May 2021 were conducted by telephone. Interviews were conducted by two authors (RA and LD), were audio-recorded and transcribed verbatim by a university-approved professional transcription company. Transcripts were checked for accuracy, anonymised, and then imported into NVivo Version 12 for analysis.

Data analysis

Framework analysis [24] and thematic analysis [25] were used to organise and analyse the data. A Framework matrix was created with headings. Notes and quotations from each

For peer review only

interview were listed under headings to summarise study data so that all authors could have an overview of key data from the whole sample.

Thematic analysis was conducted on individual interview transcripts and involved: familiarisation; generating initial codes; generating initial themes; reviewing themes; defining and naming themes; and reporting the thematic analysis [25]. Familiarisation with data was achieved through checking, reading, and re-reading of transcripts. Initial themes were generated by examining relationships between codes and common features in the data. Themes were revised and developed through discussions among authors. Data collection and analysis were conducted in tandem, and data collection continued until no new concepts or themes were found.

The authors' disciplinary backgrounds include general practice, health psychology, and health services research.

Patient and Public Involvement

Patients were not involved in the design of this qualitative study, but a key aim of this work was to understand patient and caregiver perceptions and opinions. Participants were sent a summary of the study results and invited to comment on them. Three participants provided comments on the results.

Ethics

All participants provided informed consent to participate. Ethics approval was granted by the North of Scotland Research Ethics Committee (reference 19/NS/0158).

Results

One hundred and sixty-three study invitations were sent by six general practices. Thirty-five patients and 6 caregivers (joint interviews) participated in 13 in-person face-to-face interviews, and 22 telephone interviews (21% response rate). Participant level demographics are presented in Table 1. Participants were aged between 37 and 91 years, mean age 69 years.

For peer review only

Twenty-two participants had prostate cancer and 13 had colorectal cancer (7 female, 6 male). Most participants were from areas of low socioeconomic deprivation, with 23/35 patients in deciles six through ten of the Scottish Index of Multiple Deprivation [26] (SIMD), (where one is most deprived and 10 is least deprived). Most participants were from a large urban area (45.7%), or an accessible rural area (28.6%).

Comparing the demographics of those who participated to those who were invited but did not respond, a higher proportion of non-respondents resided in areas of higher socioeconomic deprivation (42.2% of non-respondents, compared to 20% of participants). These differences were not statistically significant (see Supplementary Table 1).

Interviews lasted between 21- and 94-minutes duration, mean 44 minutes.

For peer review only

Table 1: Participant Demographics

Patient ID	Age range (years)	Sex*	Cancer site	SIMD decile†	Urban rural classification$	No. of Co-morbidities**	Carer
1	70-79	M	Prostate	10	1	0	No
2	80-89	F	Colorectal	8	1	1	No
3	90-99	F	Colorectal	7	1	2	No
4	60-69	M	Prostate	10	1	1	No
5	70-79	M	Colorectal	3	1	2	No
6	70-79	M	Prostate	5	5	2	Yes
7	60-69	M	Prostate	5	1	1	No
8	60-69	M	Prostate	7	5	1	Yes
9	70-79	M	Prostate	7	5	1	Yes
10	60-69	M	Colorectal	8	3	1	No
11	60-69	M	Prostate	7	5	0	No
12	60-69	M	Colorectal	Missing	Missing	1	No
13	60-69	F	Colorectal	9	5	0	No
14	70-79	M	Prostate	10	3	0	No
15	60-69	F	Colorectal	9	5	1	Yes
16	60-69	M	Prostate	3	1	4	No
17	60-69	M	Colorectal	7	1	1	No
18	60-69	M	Prostate	10	1	1	No
19	80-89	M	Colorectal	8	1	2	Yes
20	70-79	M	Prostate	2	1	1	No
21	70-79	M	Prostate	6	1	1	No
22	60-69	M	Prostate	9	6	4	No
23	50-59	M	Prostate	2	1	1	No
24	70-79	M	Prostate	9	5	1	No
25	70-79	M	Prostate	5	1	1	No
26	70-79	M	Prostate	10	1	5	No
27	70-79	M	Prostate	6	1	1	No
28	70-79	M	Prostate	8	6	3	No
29	50-59	M	Prostate	7	5	0	No
30	50-59	F	Colorectal	7	1	0	No
31	30-39	M	Colorectal	4	5	0	No
32	70-79	F	Colorectal	3	5	0	No
33	70-79	F	Colorectal	4	6	3	No
34	60-69	M	Prostate	5	6	0	Yes
35	60-69	M	Prostate	5	2	0	No

**Sex – M = male, F = female*
†Scottish Index of Multiple Deprivation (SIMD) is derived from postcode. One indicates most deprived areas and 10 least deprived
$Sixfold urban rural classification is also derived from Scottish postcode. 1 = Large Urban Areas, 2= Other Urban Areas, 3 – Accessible Small Towns, 4=Remote Small Towns, 5 = Accessible Rural Areas, 6 = Remote Rural Areas
***Comorbidities were self-reported by patients and were chronic conditions (not including prostate or colorectal cancer), requiring ongoing treatment or management. Other primary cancers were also included. Complications of cancer treatment (e.g. peripheral neuropathy, urinary incontinence) were not included as separate comorbidities. Individual conditions have not been listed, so as to maintain participant anonymity.*

For peer review only

Thematic Analysis

Themes and their interactions are summarised in Figure 1.

Theme 1: Getting on with the work of cancer survivorship

Participants described having little choice but to engage in the work of managing cancer and its sequelae because cancer was a threat to life. Several participants mentioned "*getting on with it*".

The work of health management after completion of treatment involved planning, organisation, problem-solving and psychological work. Individuals developed new routines, for example, pacing daily activities to manage fatigue, and pre-planning before travelling (e.g., packing necessary stoma equipment or medications).

> *[The nurse] says to me, "The difficult work will start after the operation", and I thought, "What does she mean by that? The operation must be the difficult part". But I now know by this what she means, (…) she says, "you're going to be wearing pads and everything like that". If I go to the town (…), a man doesn't seem to worry about where a toilet is. But now I need to find a toilet (…)but make sure it's got a bin.*
> Patient 7, male aged 60-69 years, prostate cancer.

Participants took active approaches to psychological adjustment after cancer. Many made comparisons with others whom they felt were "*worse off*" (e.g., more advanced cancer, other disabling chronic diseases). Some spoke of being "*determined*" or "*positive*". For many, cancer was always present in the background, and work was required to return to "*near*" normality after treatment.

Health behaviour change was a source of work for some participants, but mainly in the period immediately after cancer diagnosis. Examples included efforts to lose weight prior to prostate

For peer review only

cancer surgery to improve urinary continence or increasing exercise levels to pre-habilitate before colorectal cancer surgery.

Few participants described efforts to make dietary, exercise or other healthy behaviour changes after completion of treatment. Some considered that they already lived healthy lifestyles, whilst others considered cancer to have been cured and no longer requiring attention. Participants with both prostate and colorectal cancer suggested that there was little emphasis placed on adopting healthy behaviours by healthcare professionals after their initial treatment had finished.

> *"It took a while for my wound to heal, I'm terribly overweight (...) I was bothered because I just didn't stop eating, which I think had nothing to do with the cancer. But I couldn't stop eating and I did mention it to the oncology nurse, and she said it was probably post-stress disorder and not to worry about it."* Patient 33, female aged 70-79 years, colorectal cancer.

Theme 2: Investing time

Cancer management was time consuming during the initial treatment stages, but treatment-related work reduced over time, even when there were lasting effects or complications of treatment.

> *"Cancer management. Certainly, if you want to put it in terms of a graph, the graph has gone up steadily and quite steeply immediately post operation, but it has levelled out and is now on the way down and I'm pleased with that"* Patient 34, male aged 60-69 years, prostate cancer.

Follow-up healthcare appointments and investigations to detect recurrence were viewed positively and were reassuring. Many spoke of their gratitude that cancer was detected at a potentially curable stage and that time invested in treatment and follow-up could translate

For peer review only

into a greater amount of time to spend in the future on meaningful activities. Despite cancer management consuming considerable amounts of time, this was not perceived to be burdensome.

> *"I have a life and the treatment is actually preserving that very precious thing for me, so that means I'm going to hopefully be around for longer to be with my daughters and my grandchildren and I will be extended by this treatment (…) that to me is the most important thing."* Patient 7, male aged 60-69 years, prostate cancer.

Theme 3: the balance between empowerment and treatment burden

Participants identified individual-, disease-related-, and health system-related factors that protected against, or increased the likelihood of them experiencing treatment burden. At the level of the individual, the presence of a spouse or a strong social support network protected against treatment burden.

Caregivers/loved ones shared the practical and psychological work of cancer management, and in this sample, caregivers were keen to contribute to this work and to feel useful. One caregiver described how she felt "*helpless*" and "*lost*" when her partner experienced problematic symptoms after prostate cancer treatment. She advocated for increased involvement of the caregiver in medical encounters, and more "*direct support for carers*".

Several participants found that skills learned during their working lives helped them to manage cancer-related work. Taking action was a way to exert control over the illness.

> *"I was the Global trouble shooter of the company, I was on a plane more times than I care to remember, which I don't miss now at all. But a lot of that was problem solving (…) my work ethic was to go on and if there's a problem to be solved you just have to go out and solve it."* Participant 18, male aged 60-69 years, prostate cancer.

For peer review only

The flexibility to manage personal time (e.g., being retired), and financial security protected participants from treatment burden. Conversely, caring responsibilities, co-existing mental health problems, or challenging social circumstances increased the risk of treatment burden.

> *"losing my parents (…) it's an initial domino effect. That causes that, that causes that to cause that (…) I became homeless, that was quite dramatic (…) and then three year ago, "Oh, you've got prostate cancer" as well to boot and I went, "Oh, okay, how do you deal with this?" So I was already dealing with things, so like the cancer on top of that, "Oh, well what else is there?"'* Patient 16, male aged 60-69 years, prostate cancer.

At the disease-level, those with ongoing symptoms or managing complications related to their cancer spent more time managing their health. Several participants reflected on their experiences of supporting friends or loved ones with advanced cancer and believed that treatment workload would be much higher if their disease had been detected at a later stage.

At the health system-level, administrative problems were burdensome. Examples included lost appointments, or scans that were not arranged at the expected interval. However, participants with both prostate and colorectal cancer thought that cancer aftercare had been configured to minimise burden for them. Important factors that minimised burden included telephone access to a named specialist nurse or group of nurses; kind, caring and professional staff, close links with local cancer support organisations, timely provision of information, and a pre-determined, algorithmic treatment and follow-up plan. Participants described being on a "*pathway*", or "*in the hands of the machine*", with "*very [few] rabbit holes that you can jump down*".

For peer review only

Theme 4: comparisons and interactions between cancer and other comorbidities

Co-existing comorbidities could be more burdensome than cancer. Most participants considered that cancer had been a discrete event which had been cured, whereas other comorbidities such as Parkinson's disease and diabetes required ongoing monitoring, medication adherence, and behaviour change. One participant described making significant dietary changes to manage his hypertension but no behavioural changes after a hemicolectomy for screening detected colorectal cancer. Several participants mentioned the "invisible" nature of cancer, which made it easier to forget.

> *"I mean partly because [psoriasis is] visible and you have to physically treat it yourself, I mean it's putting on the creams and so on and dealing with the… I mean there used to be times when you would bleed (...) whereas with the prostate you don't really have to, well I haven't done anything, I haven't really done anything."* Participant 28, male aged 70-79 years, prostate cancer.

There was also a sense that cancer care was better resourced and better organised, compared to health services for dermatological, respiratory, and cardiovascular problems. One participant suggested that cancer had a high profile in the media and was favoured by politicians for financial investment compared to other chronic conditions.

The lasting effects of cancer and its treatments could interact with other comorbidities to increase treatment workload. For example, a participant with Parkinson's disease and tremor found the management of urinary incontinence more challenging.

> *"With the Parkinson's tremor (...) the organising, planning and managing the incontinence (...) is impacting me and can be more frustrating because you have to spend a lot of time on administration (...) In terms of administration, medication (...) how many of these pills have I taken, when do I need to take the next ones, what*

For peer review only

scenarios could I be in that I need to manage myself [incontinence] and if that happens how do you manage yourself if you're in a group of people and you have to take time out to sort yourself out (…) it's highly frustrating. Patient 27, male aged 70-79 years, prostate cancer.

Theme 5: the impact of treatment burden

Most of the participants in this study did not perceive themselves to be burdened by cancer aftercare. However, there were examples of treatment burden influencing cancer treatment and follow-up decisions.

An individual with type 1 diabetes explained that the workload of diabetes had influenced his decision to opt for a radical prostatectomy over more conservative options.

"I thought rather than go through [non-surgical treatment for prostate cancer] which can be through monitors and blood tests and maybe more, having to take pills or some kind of medication for your prostate rather than go for the operation, I says, "Doing insulin five times a day I think is becoming an awful lot and then having any more of a load on top of that", so that was the main reason." Patient 18, male aged 60-69 years, prostate cancer.

Another participant with a previous primary testicular cancer described "*never being away from the hospital*" when he required hospital treatments for three separate medical conditions. He became aware that his testicular cancer follow-up had not taken place when it should have:

"I just couldn't face the thought of going back through, you know, going through all these clinics and everything again, so I left it and never had any follow up for that at all." Patient 28, male aged 70-79 years, prostate cancer.

For peer review only

Discussion

Main findings

The term "burden" did not resonate strongly with most cancer survivors in this study, despite descriptions of time intensive treatment and follow up regimens and the ongoing management of problematic symptoms such as fatigue and incontinence. The notion of burden was incongruent with the gratitude that cancer survivors expressed for curative treatment. Time invested in cancer management would ultimately lead to survival and more time to spend on meaningful activities.

There were potentially modifiable factors that could make treatment burden more or less likely, including social support, financial stability, and health system configuration. Burden seemed most likely to occur in individuals with interacting comorbidities. In two patients, the desire to minimise treatment burden had influenced cancer treatment decisions or engagement with follow up.

Another key finding was that cancer was framed as a discrete episode – something invisible that had been cut out or contained and that should be consigned to the past. In this sample, cancer was not perceived to require the lifelong adherence to self-monitoring and lifestyle changes that were emphasised in conditions such as diabetes or hypertension. Cancer was not considered to be a chronic disease and none of the participants mentioned work to prevent recurrence.

Comparison with existing literature

Treatment burden has mainly been researched in multimorbidity [27]and in cardiovascular disease [13], diabetes[28], and stroke [21]. Patients are known to be burdened by "fragmented" medical care, poor communication/lack of empathy from health professionals, and inadequate information provision [14]. These problems were less evident in our sample,

For peer review only

and it was suggested by participants that cancer services are prioritised over other chronic diseases.

We identified patient-, disease-, and health system level factors that can increase or decrease treatment burden. Many of these fit well with established theory, which suggests a "cumulative complexity" of health care work [12]. Burden occurs when patient workload and demand exceeds patient capacity to undertake this work [7, 12].

An interesting question arising from this work is whether all cancers should be considered as chronic or long-term conditions. Long-term conditions are "*conditions for which there is currently no cure, and which are managed with drugs and other treatment*"[29]. Individuals in this study had been treated with curative intent. However, all participants were at risk of recurrence, many had lasting effects of treatment, and all were at slightly higher risk of second primary cancers [30, 31]. A challenge for survivorship care is how to introduce the nuance of long-term health management against the binary messages of "curable" or "incurable" that are presented during active treatment.

In this sample, opportunities to promote exercise, healthy diet and weight management were being missed but patients had capacity to take on this work.

Strengths and Limitations

This study adds new insights into burden of treatment in cancer survivors. The selected cancers encompassed a wide variety of treatment modalities, different follow-up regimes, and a spectrum of illness burdens. Over 25 hours of rich audio recorded data were generated from in depth interviews. The involvement of four authors from different backgrounds enriched the analysis with different theoretical and methodological perspectives. Results were fed-back to participants to ensure they reflected participant experiences, and that important findings were not omitted.

For peer review only

The main limitation of this work is that all participants were from a single geographical area, attending an academic teaching hospital, and most were from areas of low socioeconomic deprivation with relatively low levels of multimorbidity. Multimorbidity and socioeconomic deprivation are significant risk factors for treatment burden. This study may have underestimated the impact of treatment burden on cancer survivors. There is a paradox that those who are significantly burdened by their treatment may have less capacity to participate in research, and it is important to consider mechanisms of incentivising and including these individuals in future research.

Implications for practice and future research

Treatment burden could drive inequities in cancer outcomes. Clinicians should carefully consider what they are asking patients and their caregivers to do, and whether they have the capacity to undertake this work. Those with comorbidities in addition to cancer are particularly at risk of treatment burden. Future research might focus on innovative ways to provide accessible, structured, holistic care to multimorbid cancer survivors. There may be an increasing role for "specialist generalists"[32]. Future research should examine the relationship between treatment burden and specific, measurable outcomes after cancer such as survival, recurrence, and quality of life. Interventions might target those most at risk of treatment burden to improve their outcomes after cancer.

Conclusions

There is a continuum between empowerment and burden in cancer survivors. A cancer diagnosis serves as a strong motivator to engage in health management, but the balance between feeling empowered or experiencing burden can be shifted by individual-, disease-, and health-system factors. Treatment burden can affect engagement with and decisions about care and is an important consideration in cancer survivorship care.

For peer review only

Ethics statement

All participants provided informed consent to participate. Ethics approval was granted by the North of Scotland Research Ethics Committee (reference 19/NS/0158).

Contributorship statement

Conceptualisation – RA. Data collection – RA, LD. Data analysis – RA, LD, SM, LL. Writing original draft – RA. Revising manuscript critically – RA, LD, SM, LL.

Competing interests

The authors have no relevant financial or non-financial interests to disclose.

Funding statement

This work was funded by a Scottish Government Chief Scientist Office Senior Clinical Academic Fellowship awarded to Rosalind Adam, reference SCAF/18/02.

Data sharing statement

Participants of this study did not agree for their interview transcripts to be shared publicly, so supporting data is not available.

For peer review only

References

1. Zhu, Z., Xing, W., Zhang, X., Hu, Y., & So, W. K. W. (2020). Cancer survivors' experiences with financial toxicity: A systematic review and meta-synthesis of qualitative studies. *Psycho-oncology*, *29*(6), 945–959. https://doi.org/10.1002/pon.5361
2. Presley, C. J., Soulos, P. R., Tinetti, M., Montori, V. M., Yu, J. B., & Gross, C. P. (2017). Treatment Burden of Medicare Beneficiaries With Stage I Non-Small-Cell Lung Cancer. *Journal of oncology practice*, *13*(2), e98–e107. https://doi.org/10.1200/JOP.2016.014100
3. El-Shami, K., Oeffinger, K. C., Erb, N. L., Willis, A., Bretsch, J. K., Pratt-Chapman, M. L., … Cowens-Alvarado, R. L. (2015). American Cancer Society Colorectal Cancer Survivorship Care Guidelines. *CA: A Cancer Journal for Clinicians*, *65*(6), 427–455. https://doi.org/10.3322/caac.21286
4. Skolarus, T. A., Wolf, A. M. D., Erb, N. L., Brooks, D. D., Rivers, B. M., Underwood, W., … Cowens-Alvarado, R. L. (2014). American Cancer Society prostate cancer survivorship care guidelines. *CA: A Cancer Journal for Clinicians*, *64*(4), 225–249. https://doi.org/10.3322/caac.21234
5. van Dongen, S. I., de Nooijer, K., Cramm, J. M., Francke, A. L., Oldenmenger, W. H., Korfage, I. J., … Rietjens, J. A. (2020). Self-management of patients with advanced cancer: A systematic review of experiences and attitudes. *Palliative Medicine*, *34*(2), 160–178. https://doi.org/10.1177/0269216319883976
6. Foster, C., & Fenlon, D. (2011). Recovery and self-management support following primary cancer treatment. *British Journal of Cancer*, *105*(S1), S21–S28. https://doi.org/10.1038/bjc.2011.419
7. May, C. R., Eton, D. T., Boehmer, K., Gallacher, K., Hunt, K., MacDonald, S., … Shippee, N. (2014). Rethinking the patient: using Burden of Treatment Theory to understand the changing dynamics of illness. *BMC Health Services Research*, *14*(1), 281. https://doi.org/10.1186/1472-6963-14-281
8. Mair, F. S., & May, C. R. (2014). Thinking about the burden of treatment. *BMJ (Clinical research ed.)*, *349*, g6680. https://doi.org/10.1136/bmj.g6680 [doi]
9. Boehmer, K. R., Gallacher, K. I., Lippiett, K. A., Mair, F. S., May, C. R., & Montori, V. M. (2022). Minimally Disruptive Medicine: Progress 10 Years Later. *Mayo Clinic proceedings*, *97*(2), 210–220. https://doi.org/10.1016/j.mayocp.2021.09.003
10. The Academy of Medical Sciences. (2018). *Multimorbidity: a priority for global health research*. Retrieved from https://acmedsci.ac.uk/file-download/82222577
11. May, C. (n.d.). The Burden of Treatment and the Burden of Symptoms: the problem of workload and institutional boundaries in chronic disease. *Working Paper in Health Sciences*, *1*(9), 1–6. Retrieved from https://www.southampton.ac.uk/assets/centresresearch/documents/wphs/CRM%20the%20Burden%20of%20Treatmen.pdf
12. Shippee, N. D., Shah, N. D., May, C. R., Mair, F. S., & Montori, V. M. (2012). Cumulative complexity: a functional, patient-centered model of patient complexity can improve research and practice. *Journal of Clinical Epidemiology*, *65*(10), 1041–1051. https://doi.org/10.1016/j.jclinepi.2012.05.005
13. Gallacher, K., May, C. R., Montori, V. M., & Mair, F. S. (2011). Understanding Patients' Experiences of Treatment Burden in Chronic Heart Failure Using Normalization Process Theory. *The Annals of Family Medicine*, *9*(3), 235–243. https://doi.org/10.1370/afm.1249

For peer review only

14. Gallacher, K., Morrison, D., Jani, B., Macdonald, S., May, C. R., Montori, V. M., … Mair, F. S. (2013). Uncovering Treatment Burden as a Key Concept for Stroke Care: A Systematic Review of Qualitative Research. *PLoS Medicine*, *10*(6), e1001473. https://doi.org/10.1371/journal.pmed.1001473
15. Eton, D. T., Anderson, R. T., Cohn, W. F., Kennedy, E. M., St Sauver, J. L., Bucknell, B. J., & Ruddy, K. J. (2019). Risk factors for poor health-related quality of life in cancer survivors with multiple chronic conditions: exploring the role of treatment burden as a mediator. *Patient related outcome measures*, *10*, 89–99. https://doi.org/10.2147/PROM.S191480
16. Anderson, R. T., Eton, D. T., Camacho, F. T., Kennedy, E. M., Brenin, C. M., DeGuzman, P. B., … Cohn, W. F. (2021). Impact of comorbidities and treatment burden on general well-being among women's cancer survivors. *Journal of patient-reported outcomes*, *5*(1), 2. https://doi.org/10.1186/s41687-020-00264-z
17. Husebø, A. M. L., Dalen, I., Richardson, A., Bru, E., & Søreide, J. A. (2021). Factors influencing treatment burden in colorectal cancer patients undergoing curative surgery: A cross-sectional study. *European Journal of Cancer Care*. https://doi.org/10.1111/ecc.13437
18. El-Turk, N., Chou, M. S. H., Ting, N. C. H., Girgis, A., Vinod, S. K., Bray, V., & Dobler, C. C. (2021). Treatment burden experienced by patients with lung cancer. *PLOS ONE*, *16*(1), e0245492. https://doi.org/10.1371/journal.pone.0245492
19. Sung, H., Ferlay, J., Siegel, R. L., Laversanne, M., Soerjomataram, I., Jemal, A., & Bray, F. (2021). Global Cancer Statistics 2020: GLOBOCAN Estimates of Incidence and Mortality Worldwide for 36 Cancers in 185 Countries. *CA: a cancer journal for clinicians*, *71*(3), 209–249. https://doi.org/10.3322/caac.21660
20. Siegel, R., DeSantis, C., Virgo, K., Stein, K., Mariotto, A., Smith, T., … Ward, E. (2012). Cancer treatment and survivorship statistics, 2012. *CA: A Cancer Journal for Clinicians*, *62*(4), 220–241. https://doi.org/10.3322/caac.21149
21. Gallacher, K. I., May, C. R., Langhorne, P., & Mair, F. S. (2018). A conceptual model of treatment burden and patient capacity in stroke. *BMC family practice*, *19*(1), 9. https://doi.org/10.1186/s12875-017-0691-4
22. Murray, E., Treweek, S., Pope, C., MacFarlane, A., Ballini, L., Dowrick, C., … May, C. (2010). Normalisation process theory: a framework for developing, evaluating and implementing complex interventions. *BMC Medicine*, *8*(1), 63. https://doi.org/10.1186/1741-7015-8-63
23. Schwarzer, R., & Luszczynska, & A. (2008). The Health Action Process Approach. *European Psychologist*, *13*(2), 141–151. https://doi.org/10.1027/1016-9040.13.2.141
24. Ritchie, J., & Lewis, J. (2003). *Qualitative Research Practice A Guide for Social Science Students and Researchers* (Vol. 1). London: Sage.
25. Braun, V., & Clarke, V. (Associate P. in S. S. (n.d.). *Thematic analysis : a practical guide*. Retrieved from https://books.google.com/books/about/Thematic_Analysis.html?id=eMArEAAAQBAJ
26. Scottish Government. (n.d.). Scottish Index of Multiple Deprivation. Retrieved July 5, 2019, from https://www2.gov.scot/Topics/Statistics/SIMD
27. Duncan, P., Murphy, M., Man, M.-S., Chaplin, K., Gaunt, D., & Salisbury, C. (2018). Development and validation of the Multimorbidity Treatment Burden Questionnaire (MTBQ). *BMJ open*, *8*(4), e019413. https://doi.org/10.1136/bmjopen-2017-019413
28. Vijan, S., Hayward, R. A., Ronis, D. L., & Hofer, T. P. (2005). Brief report: the burden of diabetes therapy: implications for the design of effective patient-centered treatment regimens. *Journal of general internal medicine*, *20*(5), 479–82. https://doi.org/10.1111/j.1525-1497.2005.0117.x

For peer review only

29. The King's Fund. (n.d.). *Long-term conditions and multi-morbidity*. Retrieved from https://www.kingsfund.org.uk/projects/time-think-differently/trends-disease-and-disability-long-term-conditions-multi-morbidity
30. Chao-Yueh, F., Wen-Yen, H., Chun-Shu, L., Yu-Fu, S., Cheng-Hsiang, L., Chih-Cheng, T., … Chia-Hung, K. (2017). Risk of second primary malignancies among patients with prostate cancer: A population-based cohort study. *PloS one*, *12*(4). https://doi.org/10.1371/JOURNAL.PONE.0175217
31. Coyte, A., Morrison, D. S., & McLoone, P. (2014). Second primary cancer risk - The impact of applying different definitions of multiple primaries: Results from a retrospective population-based cancer registry study. *BMC Cancer*, *14*(1), 1–11. https://doi.org/10.1186/1471-2407-14-272/TABLES/5
32. Kyei, G. (2015). A revolution in the making: a suggested model for training NHS doctors. *British Journal of General Practice*, *65*(633), 196–197. https://doi.org/10.3399/BJGP15X684505

For peer review only

Figure 1. Themes and their interactions.

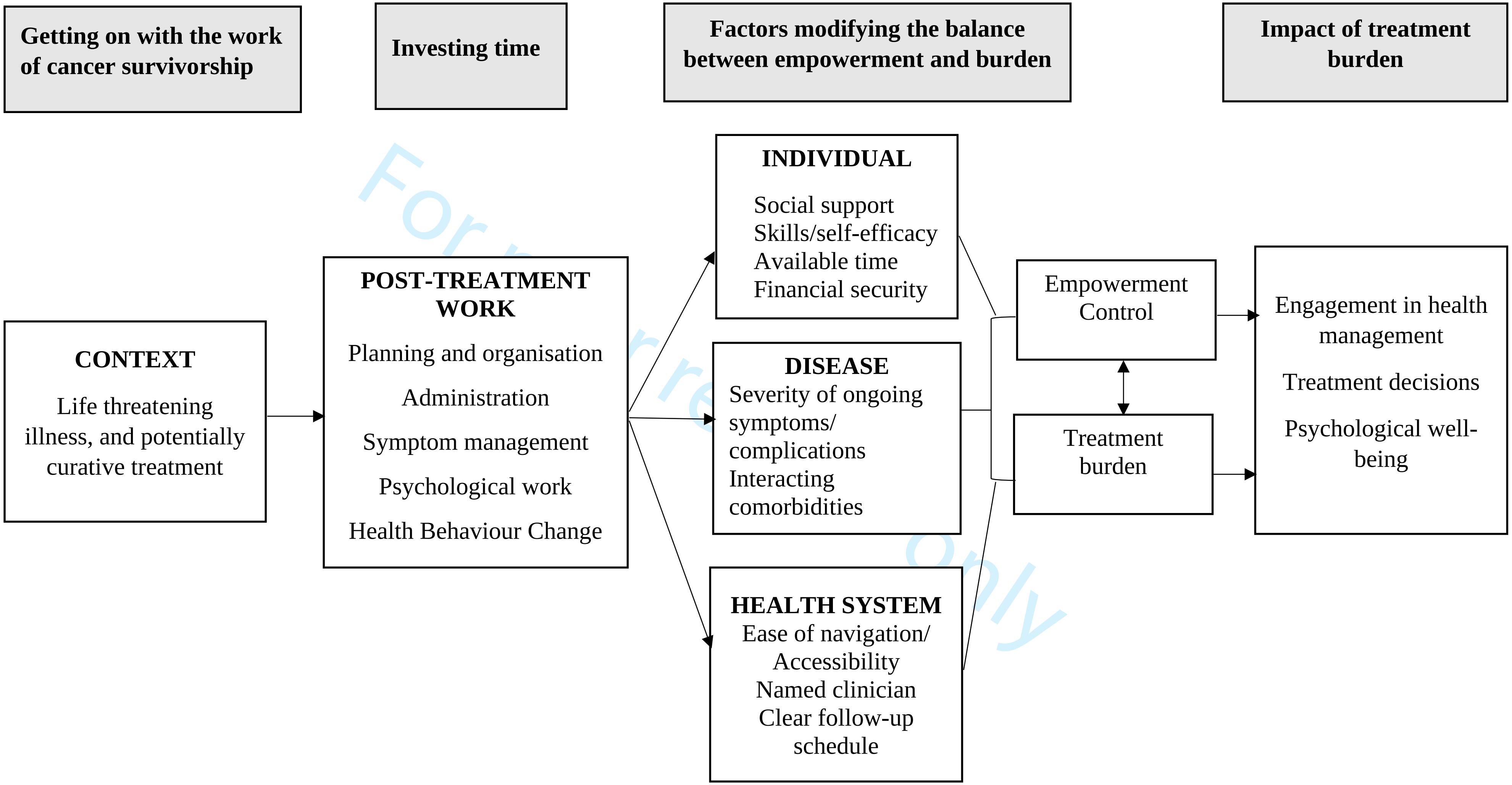

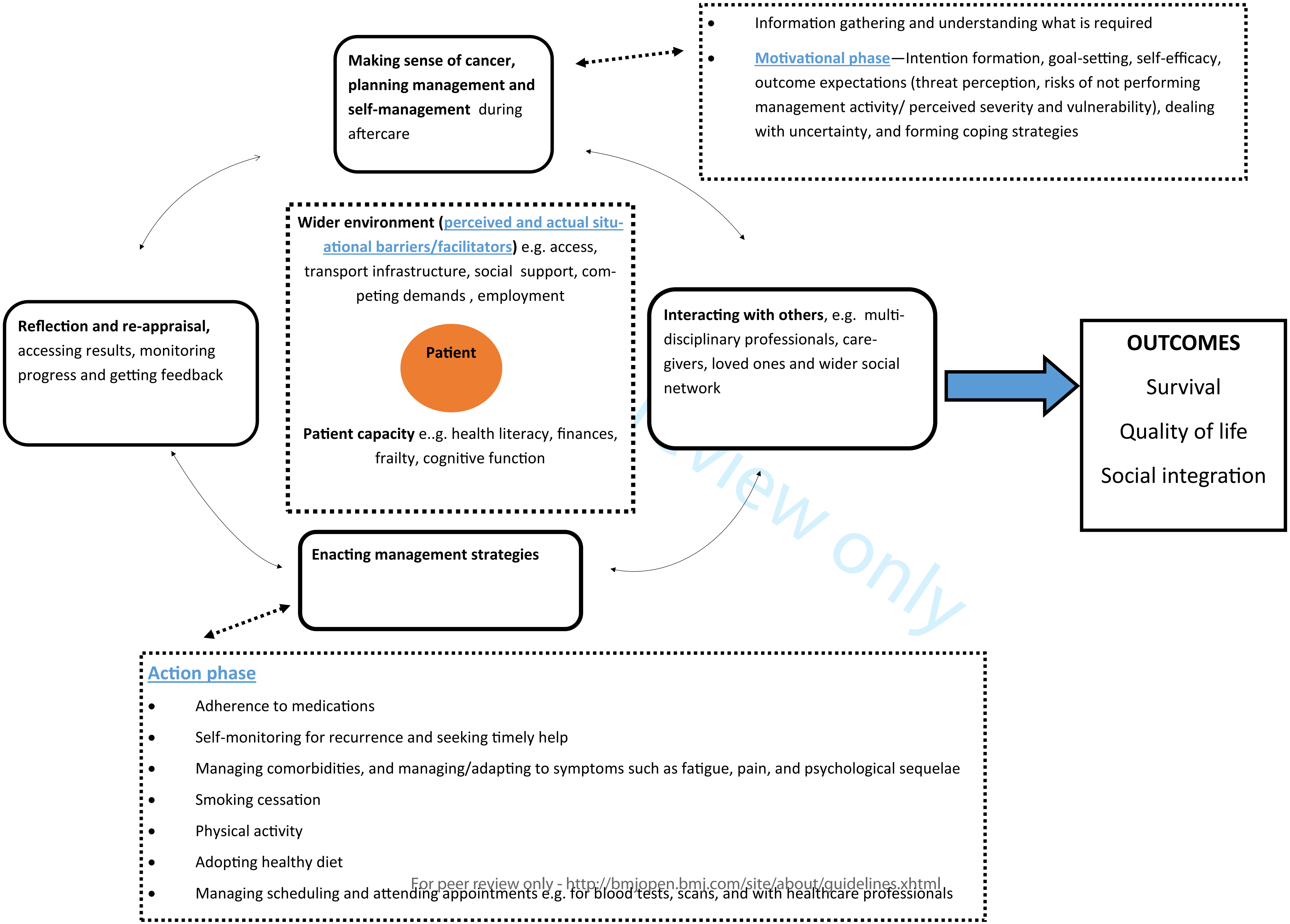

For peer review only - http://bmjopen.bmj.com/site/about/guidelines.xhtml

Supplementary Table 1. Comparisons in demographics between participants and non-participants.

	Participants (N = 35) N (%)	Non-participants invited (N = 128) N (%)	Chi-square test of independence or Fisher Exact test *p*-value
**Sex**			
Male	28 (80%)	105 (82%)	0.98
Female	7 (20%)	23 (18%)	
**Age**			
30-39	1 (2.9%)	0 (0%)	
40-49	0 (0%)	1 (0.8%)	
50-59	3 (8.6%)	16 (12.5%)	0.46
60-69	14 (40%)	40 (31.3%)	
70-79	14 (40%)	54 (42.2%)	
80+	3 (8.6%)	17 (13.3%)	
**Deprivation Index**			
1-2 (Highest deprivation)	2 (5.7%)	20 (15.6%)	
3-4	5 (14.3%)	34 (26.6%)	
5-6	7 (20%)	22 (17.2%)	0.06
7-8	11 (31.4%)	20 (15.6%)	
9-10 (Lowest deprivation)	9 (25.7%)	32 (25%)	
Missing	1 (2.9%)	0 (0%)	
**Urban Rural Classification**			
1 (Large urban)	17 (48.6%)	85 (66.4%)	
2 (Other urban)	1 (2.9%)	5 (3.9%)	
3 (Accessible small town)	2 (5.7%)	7 (5.5%)	0.09
4 (Remote small town)	0 (0%)	0 (0%)	
5 (Accessible rural)	10 (28.6%)	16 (12.5%)	
6 (Remote rural)	4 (11.4%)	15 (11.7%)	
Missing	1 (2.9%)	0 (0%)	
**Cancer type**			
Prostate	22 (62.9%)	80 (62.5%)	
Colorectal	13 (37.1%)	45 (35.2%)	1
Not specified	0	3 (2.3%)	

For peer review only

BMJ Open

Treatment burden in survivors of prostate and colorectal cancer: a qualitative interview study

Journal:	*BMJ Open*
Manuscript ID	bmjopen-2022-068997.R1
Article Type:	Original research
Date Submitted by the Author:	30-Jan-2023
Complete List of Authors:	Adam, Rosalind; University of Aberdeen, Academic Primary Care, Institute of Applied Health Sciences<br>Duncan, Lisa; University of Aberdeen, Academic Primary Care, Institute of Applied Health Sciences<br>Maclennan, Sara; University of Aberdeen, Academic Urology Unit<br>Locock, Louise; University of Aberdeen, Health Services Research Unit
**Primary Subject Heading**:	Oncology
Secondary Subject Heading:	Qualitative research
Keywords:	QUALITATIVE RESEARCH, Adult oncology < ONCOLOGY, Quality in health care < HEALTH SERVICES ADMINISTRATION & MANAGEMENT, Gastrointestinal tumours < ONCOLOGY, Urological tumours < ONCOLOGY

SCHOLARONE™
Manuscripts

I, the Submitting Author has the right to grant and does grant on behalf of all authors of the Work (as defined in the below author licence), an exclusive licence and/or a non-exclusive licence for contributions from authors who are: i) UK Crown employees; ii) where BMJ has agreed a CC-BY licence shall apply, and/or iii) in accordance with the terms applicable for US Federal Government officers or employees acting as part of their official duties; on a worldwide, perpetual, irrevocable, royalty-free basis to BMJ Publishing Group Ltd ("BMJ") its licensees and where the relevant Journal is co-owned by BMJ to the co-owners of the Journal, to publish the Work in this journal and any other BMJ products and to exploit all rights, as set out in our licence.

The Submitting Author accepts and understands that any supply made under these terms is made by BMJ to the Submitting Author unless you are acting as an employee on behalf of your employer or a postgraduate student of an affiliated institution which is paying any applicable article publishing charge ("APC") for Open Access articles. Where the Submitting Author wishes to make the Work available on an Open Access basis (and intends to pay the relevant APC), the terms of reuse of such Open Access shall be governed by a Creative Commons licence – details of these licences and which Creative Commons licence will apply to this Work are set out in our licence referred to above.

Other than as permitted in any relevant BMJ Author's Self Archiving Policies, I confirm this Work has not been accepted for publication elsewhere, is not being considered for publication elsewhere and does not duplicate material already published. I confirm all authors consent to publication of this Work and authorise the granting of this licence.

For peer review only

Treatment burden in survivors of prostate and colorectal cancer: a qualitative interview study

Dr Rosalind Adam, Academic Primary Care, Institute of Applied Health Sciences, University of Aberdeen. Room 1:020, Polwarth Building, Foresterhill, Aberdeen, AB25 2ZD. rosalindadam@abdn.ac.uk; 01224 437906. (Corresponding Author).

Miss Lisa Duncan, Academic Primary Care, Institute of Applied Health Sciences, University of Aberdeen. Room 1:023, Polwarth Building, Foresterhill, Aberdeen, AB25 2ZD.

Dr Sara MacLennan, Academic Urology Unit, Institute of Applied Health Sciences, Health Sciences Building (2nd Floor), University of Aberdeen, Foresterhill, Aberdeen, AB25 2ZD.

Professor Louise Locock, Health Services Research Unit, Institute of Applied Health Sciences, Health Sciences Building (2nd Floor), University of Aberdeen, Foresterhill, Aberdeen, AB25 2ZD.

For peer review only

Abstract

Objectives: Treatment burden is the workload of health care and the impact this has on the individual. Treatment burden is associated with poorer patient outcomes in several chronic diseases. Illness burden has been extensively studied in cancer, but little is known about treatment burden, particularly in those who have completed primary treatment for cancer. The aim of this study was to investigate treatment burden in survivors of prostate and colorectal cancer and their caregivers.

Design: Semi-structured interview study. Interviews were analysed using Framework and thematic analysis.

Setting: Participants were recruited via general practices in Northeast Scotland

Participants: Eligible participants were individuals who had been diagnosed with colorectal or prostate cancer without distant metastases within the previous five years and their caregivers. Thirty-five patients and 6 caregivers participated: 22 patients had prostate and 13 had colorectal cancers (6, male, 7 female).

Results

The term "burden" did not resonate with most survivors, who expressed gratitude that time invested in cancer care could translate into improved survival. Cancer management was time consuming, but workload reduced over time. Cancer was usually considered as a discrete episode. Individual-, disease-, and health system- factors protected against or increased treatment burden. Some factors, such as health service configuration were potentially modifiable. Multimorbidity contributed most to treatment burden and influenced treatment decisions and engagement with follow up. The presence of a caregiver protected against treatment burden, but caregivers also experienced burden.

Conclusions

For peer review only

Intensive cancer treatment and follow-up regimes do not necessarily lead to perceived burden. A cancer diagnosis serves as a strong motivator to engage in health management, but a careful balance exists between positive perceptions and burden. Treatment burden could lead to poorer cancer outcomes by influencing engagement with and decisions about care. Clinicians should ask about treatment burden and its impact, particularly in those with multimorbidity.

Trial Registration: NCT04163068; clinicaltrials.gov

Strengths and Limitations

- In-depth interviews with 41 individuals, generating rich qualitative data
- Participant feedback on the results and key messages
- The study was conducted in a single geographical area
- Participants from areas of higher social deprivation were relatively under-represented

Word count = 4284 (not including participant quotations)

Keywords – Qualitative research, adult oncology, quality in health care, gastrointestinal tumours, urological tumours

For peer review only

Introduction

Modern cancer survivorship care places physical, financial, psychosocial, and practical demands on individuals and their families [1–4]. There is a move away from hospital and clinician-led cancer care towards supported self-monitoring and self-management by patients and their families in the community [5, 6].

Treatment burden is the workload of healthcare and the impact that this work has on the individual [7]. Treatment burden is of increasing importance [8] due to ageing populations, a rising prevalence of multimorbidity [9, 10], and increased pressure on healthcare systems. Healthcare workload can encompass a wide range of tasks, including “sense-making” work [11], monitoring/managing symptoms, managing medicines, navigating the healthcare system, and changing health-related behaviours [8, 11].

Treatment burden and illness/disease burden are closely linked but conceptually different. Illness burden describes the impact of an illness on an individual, such as morbidity and mortality [12]. The actions taken to manage health and their consequences can lead to treatment burden [13–15].

Sav et al noted six key domains of treatment burden, encompassing “financial, medication, administrative, time/travel, lifestyle, and healthcare” dimensions, and “antecedents” which can influence the severity of treatment burden, such as age, gender, treatment characteristics, and disease type [16, 17]. Having good social support or a caregiver can lower treatment burden for patients [17], but caregivers can also become burdened [18, 19]. The impact of treatment burden on informal caregivers is under-researched [18].

For peer review only

Treatment burden is likely to be important in cancer survivors[1] and their caregivers. Questionnaire studies have detected high levels of treatment burden after cancer in patients, particularly in those with lower health literacy [20, 21], multiple comorbidities [20–23], and lower social support [20, 22]. One qualitative interview study investigated treatment burden in individuals who were undergoing or had recently completed treatment for lung cancer [24]. Only one caregiver participated in the study. Patients had restructured their lives to accommodate treatment-associated workload. This was accepted by patients as a necessity due to the severe and life-threatening nature of lung cancer [24]. It is unclear how treatment burden might be perceived by survivors of different types of cancer in whom the prognosis is more favourable than lung cancer.

Prostate and colorectal cancers are the third and fourth most common invasive cancers worldwide.[25] They have excellent prognoses when detected and treated early[3, 26]. Prostate and colorectal cancers were chosen to explore treatment burden in this study because they encompass a wide range of treatment modalities (surgery, radiotherapy, chemotherapy), lasting sequelae (e.g., fatigue, persistent pain, incontinence, sexual problems, and stoma management), and follow-up activities. Individuals play a key role in improving their own prognosis by self-monitoring for symptoms and attending for scans and blood tests to detect recurrence, and by adhering to diet and exercise recommendations [4, 27], and may therefore be at risk of treatment burden. Informal caregivers are key supporters of these activities [28]. The aim of this study was to investigate perceptions of treatment burden in individuals who had completed treatment for colorectal or prostate cancer and their caregivers and the impact of treatment burden on these individuals. It is held that individuals who become overburdened by the workload of health care disengage from self-management activities,

[1] The term "survivor" is used here to describe individuals living with and beyond cancer. It is a term that is widely used in research, guidelines, and charities but which can divide opinion in some individuals with cancer.

For peer review only - http://bmjopen.bmj.com/site/about/guidelines.xhtml

leading to poorer outcomes [21–23]. Treatment burden could be an important mediator of poorer outcomes in cancer survivors and patients/caregivers are best-placed to give insights into mechanisms of treatment burden and aspects that are potentially modifiable.

Methods

Setting and design

A qualitative semi-structured interview study was conducted in National Health Service (NHS) Grampian in Northeast Scotland. The NHS is a publicly funded healthcare system which is free at the point of delivery. In Grampian, cancer care is centred around a university teaching hospital in Aberdeen with academic links, and care pathways that are integrated with local cancer charities [29] Grampian had a 2011 census population of 569,061, and around one third of the population live rurally [30].

Participants and recruitment

Eligible participants were adults with a history of localised or locally advanced prostate or colorectal cancer, diagnosed within the past five years. A five-year cut off was chosen so that individuals were reflecting on recent experiences of cancer treatment and follow-up, and because many individuals with low-risk disease are discharged from follow up after five years. Participants were included if they had received any cancer treatment/management, including, and not limited to active surveillance, surgery, radiotherapy, or chemotherapy. Individuals who were undergoing or on waiting lists for chemotherapy, radiotherapy, or surgery were excluded because their experiences of treatment burden were likely to be different from individuals who were in the follow-up stages after active cancer treatment. Those with distant metastases were excluded because treatment and follow-up for these individuals would have different aims and formats of delivery.

NHS Research Scotland Primary Care (NRS Primary Care) assisted with recruitment. NRS Primary Care recruited general practices and searched electronic medical records using Read

For peer review only

codes for prostate and colorectal cancer. General practices sent invitation packs to eligible patients, and those interested in participating responded directly to the research team. Eligible patients were invited to nominate a caregiver to participate in the interview. Separate invitation letters and information sheets were included for caregivers in packs sent to patients.

Data collection and management

An interview topic guide (Supplementary File 1) was designed to ensure comprehensive coverage of the topic. A conceptual model (Supplementary Figure 1) was produced, drawing on existing literature on treatment burden in stroke [11], Normalisation Process Theory (NPT) [31], and the Health Action Process Approach (HAPA) [32]. NPT has been used to understand how individuals embed new practices within their daily life and has been a useful model through which to explore treatment burden after stroke [11]. The HAPA describes both how individuals become motivated to engage with healthcare or self-management work, and how individuals then translate this motivation into engagement with and maintenance of self-management practices over time. The HAPA integrates and extends previous behavioural theories by including a range of important constructs such as self-efficacy and intention, which can predict and explain human behaviours [33].

The conceptual model was used to inform the topic guide, which included questions about the initial diagnosis and treatment period, current self-management strategies, social support, and interactions with others. Participants were asked to reflect on the work of cancer management and any effects that managing their health had on them. The guide was used flexibly, allowing the interview to be directed by participant responses. Participants and caregivers were asked about the same topics and interviewers adapted the questions during the interview to ensure that caregiver perceptions were fully captured.

The study commenced in January 2020 and was put on hold in March 2020 during the Covid-19 pandemic, restarting in May 2021. Interviews conducted prior to March 2020 were

For peer review only

conducted face to face or by telephone, according to participant preference. All interviews undertaken after May 2021 were conducted by telephone. Interviews were conducted by two authors (RA and LD), were audio-recorded and transcribed verbatim by a university-approved professional transcription company. Transcripts were checked for accuracy, anonymised, and then imported into NVivo software (QSR International Pty Ltd. Version 12, 2018) for analysis.

Data analysis

Framework analysis [34] and thematic analysis [35] were used to organise and analyse the data. A Framework matrix was created with headings. Notes and quotations from each interview were listed under headings to summarise study data so that all authors could have an overview of key data from the whole sample.

Thematic analysis was conducted on individual interview transcripts and involved: familiarisation; generating initial codes; generating initial themes; reviewing themes; defining and naming themes; and reporting the thematic analysis [35]. Familiarisation with data was achieved through checking, reading, and re-reading of transcripts. Initial themes were generated by examining relationships between codes and common features in the data. Themes were revised and developed through discussions among authors. Data collection and analysis were conducted in tandem, and data collection continued until no new concepts or themes were found.

The authors' disciplinary backgrounds include general practice, health psychology, and health services research.

Patient and Public Involvement

Patients were not involved in the design of this qualitative study, but a key aim of this work was to understand patient and caregiver perceptions and opinions. Participants were sent a summary of the study results and invited to comment on them. Three participants replied and

For peer review only

provided general updates on their progress. None suggested any changes or additions to the results.

Ethics

All participants provided informed consent to participate. Ethics approval was granted by the North of Scotland Research Ethics Committee (reference 19/NS/0158).

Results

One hundred and sixty-three study invitations were sent by six general practices. Thirty-five patients and 6 caregivers (joint interviews) participated in 13 in-person face-to-face interviews, and 22 telephone interviews (21% response rate). Participant level demographics are presented in Table 1. Participants were aged between 37 and 91 years, mean age 69 years. Twenty-two participants had prostate cancer and 13 had colorectal cancer (7 female, 6 male). Most participants were from areas of low socioeconomic deprivation, with 23/35 patients in deciles six through ten of the Scottish Index of Multiple Deprivation [36] (SIMD), (where one is most deprived and 10 is least deprived). Most participants were from a large urban area (45.7%), or an accessible rural area (28.6%).

Comparing the demographics of those who participated to those who were invited but did not respond, a higher proportion of non-respondents resided in areas of higher socioeconomic deprivation (42.2% of non-respondents, compared to 20% of participants). These differences were not statistically significant (see Supplementary Table 1).

Interviews lasted between 21- and 94-minutes duration, mean 44 minutes.

For peer review only

Table 1: Participant Demographics

Patient ID	Age range (years)	Sex*	Cancer site	SIMD decile†	Urban rural classification$	No. of Co-morbidities**	Carer Participated
1	70-79	M	Prostate	10	1	0	No
2	80-89	F	Colorectal	8	1	1	No
3	90-99	F	Colorectal	7	1	2	No
4	60-69	M	Prostate	10	1	1	No
5	70-79	M	Colorectal	3	1	2	No
6	70-79	M	Prostate	5	5	2	Yes
7	60-69	M	Prostate	5	1	1	No
8	60-69	M	Prostate	7	5	1	Yes
9	70-79	M	Prostate	7	5	1	Yes
10	60-69	M	Colorectal	8	3	1	No
11	60-69	M	Prostate	7	5	0	No
12	60-69	M	Colorectal	Missing	Missing	1	No
13	60-69	F	Colorectal	9	5	0	No
14	70-79	M	Prostate	10	3	0	No
15	60-69	F	Colorectal	9	5	1	Yes
16	60-69	M	Prostate	3	1	4	No
17	60-69	M	Colorectal	7	1	1	No
18	60-69	M	Prostate	10	1	1	No
19	80-89	M	Colorectal	8	1	2	Yes
20	70-79	M	Prostate	2	1	1	No
21	70-79	M	Prostate	6	1	1	No
22	60-69	M	Prostate	9	6	4	No
23	50-59	M	Prostate	2	1	1	No
24	70-79	M	Prostate	9	5	1	No
25	70-79	M	Prostate	5	1	1	No
26	70-79	M	Prostate	10	1	5	No
27	70-79	M	Prostate	6	1	1	No
28	70-79	M	Prostate	8	6	3	No
29	50-59	M	Prostate	7	5	0	No
30	50-59	F	Colorectal	7	1	0	No
31	30-39	M	Colorectal	4	5	0	No
32	70-79	F	Colorectal	3	5	0	No
33	70-79	F	Colorectal	4	6	3	No
34	60-69	M	Prostate	5	6	0	Yes
35	60-69	M	Prostate	5	2	0	No

**Sex – M = male, F = female*
†Scottish Index of Multiple Deprivation (SIMD) is derived from postcode. One indicates most deprived areas and 10 least deprived
$Sixfold urban rural classification is also derived from Scottish postcode. 1 = Large Urban Areas, 2= Other Urban Areas, 3 – Accessible Small Towns, 4=Remote Small Towns, 5 = Accessible Rural Areas, 6 = Remote Rural Areas
***Comorbidities were self-reported by patients and were chronic conditions (not including prostate or colorectal cancer), requiring ongoing treatment or management. Other primary cancers were also included. Complications of cancer treatment (e.g. peripheral neuropathy, urinary incontinence) were not included as separate comorbidities. Individual conditions have not been listed, so as to maintain participant anonymity.*

For peer review only

Thematic Analysis

Themes and their interactions are summarised in Figure 1.

Theme 1: Getting on with the work of cancer survivorship

Participants described having little choice but to engage in the work of managing cancer and its sequelae because cancer was a threat to life. Several participants mentioned "*getting on with it*".

The work of health management after completion of treatment involved planning, organisation, problem-solving and psychological work. Individuals developed new routines, for example, pacing daily activities to manage fatigue, and pre-planning before travelling (e.g., packing necessary stoma equipment or medications).

> *[The nurse] says to me, "The difficult work will start after the operation", and I thought, "What does she mean by that? The operation must be the difficult part". But I now know by this what she means, (...) she says, "you're going to be wearing pads and everything like that". If I go to the town (...), a man doesn't seem to worry about where a toilet is. But now I need to find a toilet (...) but make sure it's got a bin.* Patient 7, male aged 60-69 years, prostate cancer.

Participants took active approaches to psychological adjustment after cancer. Many made comparisons with others whom they felt were "*worse off*" (e.g., more advanced cancer, other disabling chronic diseases). Some spoke of being "*determined*" or "*positive*". For many, cancer was always present in the background, and work was required to return to "*near*" normality after treatment.

> *"I do feel that I totally lost two years of my life, because I think it took that time to get through the operation, get back to feeling normal (...) I think everything had changed about my life, in a way it had stopped and I've started a new life now (...) you think*

For peer review only

things will never be normal again, but you find a new normal." Participant 15, female aged 60-69 years, colorectal cancer.

Health behaviour change was a source of work for some participants, but mainly in the period immediately after cancer diagnosis. Examples included efforts to lose weight prior to prostate cancer surgery to improve urinary continence or increasing exercise levels to pre-habilitate before colorectal cancer surgery.

Few participants described efforts to make dietary, exercise or other healthy behaviour changes after completion of treatment. Some considered that they already lived healthy lifestyles, whilst others considered cancer to have been cured and no longer requiring attention. None of the participants spoke of specific efforts to reduce their risk of cancer recurrence. Participants with both prostate and colorectal cancer suggested that there was little emphasis placed on adopting healthy behaviours by healthcare professionals after their initial treatment had finished.

"It took a while for my wound to heal, I'm terribly overweight (...) I was bothered because I just didn't stop eating, which I think had nothing to do with the cancer. But I couldn't stop eating and I did mention it to the oncology nurse, and she said it was probably post-stress disorder and not to worry about it." Patient 33, female aged 70-79 years, colorectal cancer.

Theme 2: Investing time

Cancer management was time consuming during the initial treatment stages, but treatment-related work reduced over time, even when there were lasting effects or complications of treatment.

"Cancer management. Certainly, if you want to put it in terms of a graph, the graph has gone up steadily and quite steeply immediately post operation, but it has levelled

For peer review only

out and is now on the way down and I'm pleased with that" Patient 34, male aged 60-69 years, prostate cancer.

Follow-up healthcare appointments and investigations to detect recurrence were viewed positively and were reassuring. Many spoke of their gratitude that cancer was detected at a potentially curable stage and that time invested in treatment and follow-up could translate into a greater amount of time to spend in the future on meaningful activities. Despite cancer management consuming considerable amounts of time, this was not perceived to be burdensome.

"I have a life and the treatment is actually preserving that very precious thing for me, so that means I'm going to hopefully be around for longer to be with my daughters and my grandchildren and I will be extended by this treatment (...) that to me is the most important thing." Patient 7, male aged 60-69 years, prostate cancer.

Theme 3: factors modifying the experience of treatment burden

Participants identified individual-, disease-related-, and health system-related factors that protected against, or increased the likelihood of them experiencing treatment burden. At the level of the individual, the presence of a caregiver or a good social support network lessened the treatment burden associated with cancer. Spouses and other family members shared in the practical and psychological work of cancer management, from driving to and being present at appointments and undertaking domestic tasks, to giving input into treatment decisions and providing emotional support. During interviews, caregiver participants used the terms "us" and "we" when they spoke of diagnostic, treatment, and aftercare events, indicating that the experiences had happened to both parties. Patient/caregiver dyads described a team approach to cancer management.

For peer review only

> *"The worst bit was the colonoscopy that you had to get. That was the last follow up after all the procedures we had been through (…later in same interview) The two of you work together and we were lucky [to] both be here at the same time."* Patient 15's husband/caregiver.

At the individual-level, participants found that skills learned during their working lives helped them to manage cancer-related work. Taking action was a way to exert control over the illness.

> *"I was the Global trouble shooter of the company, I was on a plane more times than I care to remember, which I don't miss now at all. But a lot of that was problem solving (…) my work ethic was to go on and if there's a problem to be solved you just have to go out and solve it."* Participant 18, male aged 60-69 years, prostate cancer.

The flexibility to manage personal time (e.g., being retired), and financial security protected participants from treatment burden. Conversely, caring responsibilities, co-existing mental health problems, or challenging social circumstances increased the risk of treatment burden.

> *"losing my parents (…) it's an initial domino effect. That causes that, that causes that to cause that (…) I became homeless, that was quite dramatic (…) and then three year ago, "Oh, you've got prostate cancer" as well to boot and I went, "Oh, okay, how do you deal with this?" So I was already dealing with things, so like the cancer on top of that, "Oh, well what else is there?"'* Patient 16, male aged 60-69 years, prostate cancer.

At the disease-level, those with ongoing symptoms or managing complications related to their cancer or comorbidities spent more time managing their health. Several participants reflected on their experiences of supporting friends or loved ones with advanced cancer and

For peer review only

believed that treatment workload would be much higher if their disease had been detected at a later stage.

At the health system-level, administrative problems were burdensome. Examples included lost appointments, or scans that were not arranged at the expected interval. However, participants with both prostate and colorectal cancer thought that cancer aftercare had been configured to minimise burden for them. Important factors that minimised burden included telephone access to a named specialist nurse or group of nurses; kind, caring and professional staff, close links with local cancer support organisations, timely provision of information, and a pre-determined, algorithmic treatment and follow-up plan. Participants described being on a "*pathway*" (patient 24), or "*in the hands of the machine*" (patient 24), with "*very [few] rabbit holes that you can jump down*"(patient 22).

Theme 4: comparisons and interactions between cancer and other comorbidities

Co-existing comorbidities could be more burdensome than cancer. Most participants considered that cancer had been a discrete event which had been cured, whereas other comorbidities such as Parkinson's disease and diabetes required ongoing monitoring, medication adherence, and behaviour change. One participant described making significant dietary changes to manage his hypertension but no behavioural changes after a hemicolectomy for screening detected colorectal cancer. Several participants mentioned the "invisible" nature of cancer, which made it easier to forget.

> *"I mean partly because [psoriasis is] visible and you have to physically treat it yourself, I mean it's putting on the creams and so on and dealing with the… I mean there used to be times when you would bleed (…) whereas with the prostate you don't really have to, well I haven't done anything, I haven't really done anything."*
> Participant 28, male aged 70-79 years, prostate cancer.

For peer review only

There was also a sense that cancer care was better resourced and better organised, compared to health services for dermatological, respiratory, and cardiovascular problems. Two participants suggested that cancer had a high profile in the media and one suggested that cancer was favoured by politicians for financial investment compared to other chronic conditions.

The lasting effects of cancer and its treatments could interact with other comorbidities to increase treatment workload. For example, a participant with Parkinson's disease and tremor found the management of urinary incontinence more challenging.

> *"With the Parkinson's tremor (…) the organising, planning and managing the incontinence (…) is impacting me and can be more frustrating because you have to spend a lot of time on administration (…) In terms of administration, medication (…) how many of these pills have I taken, when do I need to take the next ones, what scenarios could I be in that I need to manage myself [incontinence] and if that happens how do you manage yourself if you're in a group of people and you have to take time out to sort yourself out (…) it's highly frustrating.* Patient 27, male aged 70-79 years, prostate cancer.

Theme 5: caring for the caregiver

There was not always a clear distinction between patient and caregiver roles. One caregiver spoke of their own experiences of having cancer and two patient participants were the main caregivers for their wives (who did not participate in this study). These patients experienced the multiplicative burden of managing their own health after cancer whilst continuing to provide care for their loved one. Both spoke of their role as a caregiver being their main priority.

For peer review only

"The weight gain irritates me, and I try to put more exercise in with the indoor cycle, but if I do an indoor cycle for an hour something else has to drop out of my routine in the day, I just don't have the energy to do that, particularly you know, I have the commitment to my wife's care, and I presume that's number one." Patient 14, male aged 70-79 years, prostate cancer.

The team approach to cancer management (described in theme 3) affected the caregiver. Some caregivers had taken early retirement or rearranged their working lives to accommodate caring. One caregiver (daughter of patient 19) described driving 40 miles every day to support her parents. Caregivers shared the emotional burden of cancer.

Patient: *I think it was the first CT scan, and I came out and I said to [my husband], "You're looking terrible, you look worried". He said, "Well I'm sitting here but I'm in there with you". It's affecting him just the same, maybe worse at times".*

Husband: *You're still sitting there waiting (...) your mind's in overtime just wondering how everything's going and hoping everything's okay, so just it affects you both in a different way.* Patient 15, female aged 60-69 years, colorectal cancer and her husband.

Many men with prostate cancer spoke about the impact that loss of libido and erectile dysfunction had on their relationships. In interviews involving caregiver dyads, sexual dysfunction was acknowledged as "*a source of sadness*" (patient 8), and something which had a continuing effect on both the patient and the caregiver.

"We've got the continuing difficulty of my erectile dysfunction (....) We're able to talk openly about it. I don't have the spark anymore, the sensual spark, (...) so that [problem] continues to run for both of us." Patient 34, male aged 60-69 years, prostate cancer.

For peer review only

Caregivers spoke of additional difficulties being on the “side lines” (wife of patient 8) during cancer care, and that, despite playing a key role in treatment decisions and enabling successful recovery, they had limited access to advice or individualised support.

> *“I do think it was frustrating (...) that there wasn’t a direct contact that I could’ve had as the partner, the caregiver because there were some quite concerning moments when he had different reactions (...) it would’ve been nice to actually speak directly with a medical professional and get some advice. So I did feel a bit lost regarding that (...) and a bit helpless that I couldn’t advise and couldn’t really help him”.* Partner of patient 34.

Theme 6: the impact of treatment burden

Most of the participants in this study did not perceive themselves to be burdened by cancer aftercare. However, there were three examples of treatment burden influencing cancer treatment and follow-up decisions.

An individual with type 1 diabetes explained that the workload of diabetes had influenced his decision to opt for a radical prostatectomy over more conservative options.

> *“I thought rather than go through [non-surgical treatment for prostate cancer] which can be through monitors and blood tests and maybe more, having to take pills or some kind of medication for your prostate rather than go for the operation, I says, “Doing insulin five times a day I think is becoming an awful lot and then having any more of a load on top of that”, so that was the main reason.”* Patient 18, male aged 60-69 years, prostate cancer.

Another participant with previous primary testicular cancer described “*never being away from the hospital*” when he required hospital treatments for three separate medical conditions.

For peer review only

He became aware that his testicular cancer follow-up had not taken place when it should have:

> *"I just couldn't face the thought of going back through, you know, going through all these clinics and everything again, so I left it and never had any follow up for that at all."* Patient 28, male aged 70-79 years, prostate cancer.

For this participant, perceptions about treatment burden changed over time. During active surveillance for prostate cancer, his other comorbidities were better controlled, and he was older and retired from work. He reflected that, despite spending considerable time travelling to appointments, he did not perceive prostate cancer surveillance to be burdensome.

> *"We stay out in the countryside so every trip into the hospital takes so much longer, it's like if you're going to the hospital for an appointment that's really your day taken up. (...) I don't regard it as an inconvenience, I don't resent it, it doesn't get me annoyed or anything like that, I just think, "Oh, it's something I've got to do". Yeah, time passes so quick when you're older, it won't be long before you're back home anyway."* Patient 28, male aged 70-79 years, prostate cancer.

Two participants (patients 16 and 28) were undergoing active surveillance for prostate cancer and reflected on the invisible nature of the disease. For participant 28, this made it easier to forget about than other comorbidities (see theme four) but participant 16 experienced psychological burden arising from an invisible disease that could only be monitored through medical tests.

> *The cancer isn't something that's like a broken leg ... It's just something you try to imagine or picture as a fault in the system (...) the only answer to the prostate cancer is biopsies, MRI scans and PSAs.... it's monitoring that the cancer itself if it's on the move or if it's getting bigger or I don't know if it would ever disappear without*

For peer review only

> *treatment. But whether that is contributing to my mental attitude or sometimes the way I think or feel, it's something that's just not very good, not very nice, not nice at all.* Patient 16, male aged 60-69 years, prostate cancer.

The same individual found prostate biopsies to be "*intrusive*" and "*traumatic*" and started to disengage with monitoring.

> *I haven't seen a doctor, I don't want to see a doctor. (...) it was traumatic enough to say that I'm just sick of this idea of giving biopsies, seeing doctors. And I even chickened out, I don't know if that's the word, I even missed giving a PSA on the three monthly after May"* Patient 16, male aged 60-69 years, prostate cancer.

Discussion

Main findings

The term "burden" did not resonate strongly with most cancer survivors in this study, despite descriptions of time intensive treatment and follow up regimens and the ongoing management of problematic symptoms such as fatigue and incontinence. The notion of burden was incongruent with the gratitude that cancer survivors expressed for curative treatment. Time invested in cancer management would ultimately lead to survival and more time to spend on meaningful activities.

Cancer was framed as a discrete episode – something invisible that had been cut out or contained and that should be consigned to the past. In this sample, cancer was not perceived to require the lifelong adherence to self-monitoring and lifestyle changes that were emphasised in conditions such as diabetes or hypertension. Cancer was not considered to be a chronic disease and none of the participants mentioned work to prevent recurrence.

There were potentially modifiable factors that could make treatment burden more or less likely, including social support, financial stability, and health system configuration. In this

For peer review only

sample, the most important factors were interacting comorbidities, which increased burden, and the presence of a caregiver, which reduced treatment burden. Caregivers shared in all aspects of the work of cancer survivorship care, and in the emotional and practical consequences of this work such as experiencing worry and disruption to working lives. Despite few patients perceiving cancer management to be burdensome, there were three examples in which treatment burden had influenced cancer treatment decisions or disengagement with follow up. Interestingly, individuals with similar treatment regimens had very different perceptions of burden, and perceptions of treatment burden could change over time. Disengagement with cancer monitoring is a cause for concern and is a feasible mechanism through which treatment burden could negatively affect cancer outcomes.

Comparison with existing literature

Treatment burden has mainly been researched in multimorbidity [37]and in cardiovascular disease [38], diabetes[39], and stroke [11]. Patients are known to be burdened by "fragmented" medical care, poor communication/lack of empathy from health professionals, and inadequate information provision [40]. These problems were less evident in our sample, and it was suggested by participants that cancer services are prioritised over other chronic diseases because of their prominence in the media and public eye.

A recent scoping review found that financial burden, time/travel burden and medication burden were the most prominent dimensions of treatment burden in older individuals with cancer [16]. Several studies have focused on individual factors which can contribute to treatment burden and diminished quality of life, such as financial toxicity, time spent on cancer treatments, and the burden of adhering to medications [15, 41, 42]. In our study, treatment burden was considered as a multidimensional concept. Financial and time/travel burden were not significant problems in our participants, despite almost half the sample

For peer review only

living in rural areas. This emphasises that burden is a subjective perception that is influenced by multiple interacting factors.

We identified patient-, disease-, and health system level factors that can increase or decrease treatment burden. Many of these fit well with established theory, which suggests a "cumulative complexity" of health care work [43]. Burden occurs when patient workload and demand exceeds patient capacity to undertake this work [7, 43]. A prominent difference in our sample of individuals with good prognosis cancers compared to other chronic diseases like stroke or heart failure was that the prospect of cure and extending life enhanced patients psychological and motivational capacity to engage with health-related work. This diminished the perception of burden.

An interesting question arising from this work is whether all cancers should be considered as chronic or long-term conditions. Long-term conditions are "*conditions for which there is currently no cure, and which are managed with drugs and other treatment*"[44]. Individuals in this study had been treated with curative intent. However, all participants were at risk of recurrence, many had lasting effects of treatment, and all were at slightly higher risk of second primary cancers [45, 46]. A challenge for survivorship care is how to introduce the nuance of long-term health management against the binary messages of "curable" or "incurable" that are presented during active treatment.

In this sample, opportunities to promote exercise, healthy diet and weight management were being missed but patients had capacity to take on this work.

Strengths and Limitations

This study is one of the first to specifically investigate burden of treatment and its impact in survivors of good prognosis cancers and their caregivers. It adds granular, mechanistic details to previous quantitative observations that comorbidities and social support can influence patient-ratings of treatment burden [20, 22]. The selected cancers encompassed a wide variety

For peer review only

of treatment modalities, different follow-up regimes, and a spectrum of illness burdens. Over 25 hours of rich audio recorded data were generated from in depth interviews. The involvement of four authors from different backgrounds enriched the analysis with different theoretical and methodological perspectives. Results were fed-back to participants to ensure they reflected participant experiences, and that important findings were not omitted.

There are important limitations to note. As prostate cancer is a male cancer, men are over-represented in this sample. Gender is an important antecedent of treatment burden [17], but gender issues were not specifically probed during interviews. Men gave detailed and open accounts of gender-specific problems relating to hormonal treatment and prostate surgery, such as erectile dysfunction, loss of libido and urinary symptoms. However, it is possible that broader issues relating to gender and treatment burden have been missed. Some research has suggested that women may experience more treatment burden than men [22, 47–49], but the contribution of gender and identity to treatment burden is under-researched [50]. The addition of a female cancer in this study might have highlighted important gender-related differences in treatment burden experiences.

Only six caregivers were recruited, five of whom were female. Despite this, caregivers contributed meaningful insights into their role and experiences of treatment burden, and it became clear that patient and caregiver roles were not mutually exclusive. In future research it would be important to target caregivers through specific channels of recruitment. Interviewing caregivers on their own could also highlight aspects of their experience that they might be less likely to discuss with their loved one.

All participants were from a single geographical area, attending an academic teaching hospital, and most were from areas of low socioeconomic deprivation with relatively low levels of multimorbidity. Multimorbidity and socioeconomic deprivation are significant risk factors for treatment burden. This study may have underestimated the impact of treatment

For peer review only

burden on cancer survivors. There is a paradox that those who are significantly burdened by their treatment may have less capacity to participate in research, and it is important to consider mechanisms of incentivising and including these individuals in future research.

Implications for cancer survivors, practice, and future research

Cancer survivors can be reassured that treatment burden tends to decrease over time after active treatment, and that most of the individuals in this study were not burdened by health-related workload. Nevertheless, there were indications that treatment burden could drive inequities in cancer outcomes, particularly in individuals with limited social support and concurrent comorbidities. Clinicians should carefully consider what they are asking patients and their caregivers to do, and whether they have the capacity to undertake this work. Future research might focus on innovative ways to provide accessible, structured, holistic care to multimorbid cancer survivors. There may be an increasing role for "specialist generalists"[51]. Future research should examine the relationship between treatment burden and specific, measurable outcomes after cancer such as survival, recurrence, and quality of life. Interventions might target those most at risk of treatment burden to improve their outcomes after cancer.

Conclusions

There is a continuum between positive perceptions of health-related work and burden in cancer survivors. A cancer diagnosis serves as a strong motivator to engage in health management, and perceptions about burden can be shifted by individual-, disease-, and health-system factors. Treatment burden can affect engagement with and decisions about care and is an important consideration in cancer survivorship care.

Ethics statement

For peer review only

All participants provided informed consent to participate. Ethics approval was granted by the North of Scotland Research Ethics Committee (reference 19/NS/0158).

Acknowledgements

The authors would like to thank the participants who volunteered their time to give detailed accounts of their experiences. We would also like to thank the National Health Service Research Scotland Primary Care Network, who facilitated patient recruitment via general practices. We thank the general practices who participated.

Contributorship statement

Conceptualisation – RA. Data collection – RA, LD. Data analysis – RA, LD, SM, LL. Writing original draft – RA. Revising manuscript critically – RA, LD, SM, LL.

Competing interests

The authors have no relevant financial or non-financial interests to disclose.

Funding statement

This work was funded by a Scottish Government Chief Scientist Office Senior Clinical Academic Fellowship awarded to Rosalind Adam, reference SCAF/18/02. The funder did not play a role in data collection, interpretation, or reporting.

Data sharing statement

Participants of this study did not agree for their interview transcripts to be shared publicly, so supporting data is not available.

For peer review only

References

1. Zhu, Z., Xing, W., Zhang, X., Hu, Y., & So, W. K. W. (2020). Cancer survivors' experiences with financial toxicity: A systematic review and meta-synthesis of qualitative studies. *Psycho-oncology*, *29*(6), 945–959. https://doi.org/10.1002/pon.5361
2. Presley, C. J., Soulos, P. R., Tinetti, M., Montori, V. M., Yu, J. B., & Gross, C. P. (2017). Treatment Burden of Medicare Beneficiaries With Stage I Non-Small-Cell Lung Cancer. *Journal of oncology practice*, *13*(2), e98–e107. https://doi.org/10.1200/JOP.2016.014100
3. El-Shami, K., Oeffinger, K. C., Erb, N. L., Willis, A., Bretsch, J. K., Pratt-Chapman, M. L., … Cowens-Alvarado, R. L. (2015). American Cancer Society Colorectal Cancer Survivorship Care Guidelines. *CA: A Cancer Journal for Clinicians*, *65*(6), 427–455. https://doi.org/10.3322/caac.21286
4. Skolarus, T. A., Wolf, A. M. D., Erb, N. L., Brooks, D. D., Rivers, B. M., Underwood, W., … Cowens-Alvarado, R. L. (2014). American Cancer Society prostate cancer survivorship care guidelines. *CA: A Cancer Journal for Clinicians*, *64*(4), 225–249. https://doi.org/10.3322/caac.21234
5. van Dongen, S. I., de Nooijer, K., Cramm, J. M., Francke, A. L., Oldenmenger, W. H., Korfage, I. J., … Rietjens, J. A. (2020). Self-management of patients with advanced cancer: A systematic review of experiences and attitudes. *Palliative Medicine*, *34*(2), 160–178. https://doi.org/10.1177/0269216319883976
6. Foster, C., & Fenlon, D. (2011). Recovery and self-management support following primary cancer treatment. *British Journal of Cancer*, *105*(S1), S21–S28. https://doi.org/10.1038/bjc.2011.419
7. May, C. R., Eton, D. T., Boehmer, K., Gallacher, K., Hunt, K., MacDonald, S., … Shippee, N. (2014). Rethinking the patient: using Burden of Treatment Theory to understand the changing dynamics of illness. *BMC Health Services Research*, *14*(1), 281. https://doi.org/10.1186/1472-6963-14-281
8. Mair, F. S., & May, C. R. (2014). Thinking about the burden of treatment. *BMJ (Clinical research ed.)*, *349*, g6680. https://doi.org/10.1136/bmj.g6680 [doi]
9. Boehmer, K. R., Gallacher, K. I., Lippiett, K. A., Mair, F. S., May, C. R., & Montori, V. M. (2022). Minimally Disruptive Medicine: Progress 10 Years Later. *Mayo Clinic proceedings*, *97*(2), 210–220. https://doi.org/10.1016/j.mayocp.2021.09.003
10. The Academy of Medical Sciences. (2018). *Multimorbidity: a priority for global health research*. Retrieved from https://acmedsci.ac.uk/file-download/82222577
11. Gallacher, K. I., May, C. R., Langhorne, P., & Mair, F. S. (2018). A conceptual model of treatment burden and patient capacity in stroke. *BMC family practice*, *19*(1), 9. https://doi.org/10.1186/s12875-017-0691-4
12. McGuire, T., Wells, K. B., Bruce, M. L., Miranda, J., Scheffler, R., Durham, M., … Lewis, L. (2002). Burden of Illness. *Mental Health Services Research*, *4*(4), 179–185. https://doi.org/10.1023/A:1020956313890
13. Henry, D. H., Viswanathan, H. N., Elkin, E. P., Traina, S., Wade, S., & Cella, D. (2008). Symptoms and treatment burden associated with cancer treatment: results from a cross-sectional national survey in the U.S. *Supportive Care in Cancer*, *16*(7), 791–801. https://doi.org/10.1007/s00520-007-0380-2
14. Adam, R., de Bruin, M., Burton, C. D., Bond, C. M., Giatsi Clausen, M., & Murchie, P. (2017). What are the current challenges of managing cancer pain and could digital technologies help? *BMJ supportive & palliative care*, bmjspcare-2016-001232. https://doi.org/10.1136/bmjspcare-2016-001232

For peer review only

15. Abrams, H. R., Durbin, S., Huang, C. X., Johnson, S. F., Nayak, R. K., Zahner, G. J., & Peppercorn, J. (2021). Financial toxicity in cancer care: origins, impact, and solutions. *Translational Behavioral Medicine*, *11*(11), 2043–2054. https://doi.org/10.1093/tbm/ibab091
16. Sav, A., McMillan, S. S., & Akosile, A. (2021). Burden of Treatment among Elderly Patients with Cancer: A Scoping Review. *Healthcare (Basel, Switzerland)*, *9*(5). https://doi.org/10.3390/healthcare9050612
17. Sav, A., King, M. A., Whitty, J. A., Kendall, E., McMillan, S. S., Kelly, F., … Wheeler, A. J. (2015). Burden of treatment for chronic illness: a concept analysis and review of the literature. *Health Expectations*, *18*(3), 312–324. https://doi.org/10.1111/hex.12046
18. Sav, A., Thomas, S. T., Cardona, M., Michaleff, Z. A., & Dobler, C. C. (2022). Treatment Burden Discussion in Clinical Encounters: Priorities of COPD Patients, Carers and Physicians. *International Journal of Chronic Obstructive Pulmonary Disease*, *Volume 17*, 1929–1942. https://doi.org/10.2147/COPD.S366412
19. Kent, E. E., Rowland, J. H., Northouse, L., Litzelman, K., Chou, W.-Y. S., Shelburne, N., … Huss, K. (2016). Caring for caregivers and patients: Research and clinical priorities for informal cancer caregiving. *Cancer*, *122*(13), 1987–1995. https://doi.org/10.1002/cncr.29939
20. Eton, D. T., Anderson, R. T., Cohn, W. F., Kennedy, E. M., St Sauver, J. L., Bucknell, B. J., & Ruddy, K. J. (2019). Risk factors for poor health-related quality of life in cancer survivors with multiple chronic conditions: exploring the role of treatment burden as a mediator. *Patient related outcome measures*, *10*, 89–99. https://doi.org/10.2147/PROM.S191480
21. Anderson, R. T., Eton, D. T., Camacho, F. T., Kennedy, E. M., Brenin, C. M., DeGuzman, P. B., … Cohn, W. F. (2021). Impact of comorbidities and treatment burden on general well-being among women's cancer survivors. *Journal of patient-reported outcomes*, *5*(1), 2. https://doi.org/10.1186/s41687-020-00264-z
22. Husebø, A. M. L., Dalen, I., Richardson, A., Bru, E., & Søreide, J. A. (2021). Factors influencing treatment burden in colorectal cancer patients undergoing curative surgery: A cross-sectional study. *European Journal of Cancer Care*. https://doi.org/10.1111/ecc.13437
23. Mian, H. S., Fiala, M. A., & Wildes, T. M. (2021). Burden of Treatment Among Older Adults With Newly Diagnosed Multiple Myeloma. *Clinical Lymphoma Myeloma and Leukemia*, *21*(2), e152–e159. https://doi.org/10.1016/J.CLML.2020.09.010
24. El-Turk, N., Chou, M. S. H., Ting, N. C. H., Girgis, A., Vinod, S. K., Bray, V., & Dobler, C. C. (2021). Treatment burden experienced by patients with lung cancer. *PLOS ONE*, *16*(1), e0245492. https://doi.org/10.1371/journal.pone.0245492
25. Sung, H., Ferlay, J., Siegel, R. L., Laversanne, M., Soerjomataram, I., Jemal, A., & Bray, F. (2021). Global Cancer Statistics 2020: GLOBOCAN Estimates of Incidence and Mortality Worldwide for 36 Cancers in 185 Countries. *CA: a cancer journal for clinicians*, *71*(3), 209–249. https://doi.org/10.3322/caac.21660
26. Siegel, R., DeSantis, C., Virgo, K., Stein, K., Mariotto, A., Smith, T., … Ward, E. (2012). Cancer treatment and survivorship statistics, 2012. *CA: A Cancer Journal for Clinicians*, *62*(4), 220–241. https://doi.org/10.3322/caac.21149
27. van Blarigan, E. L., & Meyerhardt, J. A. (2015). Role of Physical Activity and Diet After Colorectal Cancer Diagnosis. *Journal of Clinical Oncology*, *33*(16), 1825–1834. https://doi.org/10.1200/JCO.2014.59.7799
28. Lund, L., Ross, L., Petersen, M. A., & Groenvold, M. (2014). Cancer caregiving tasks and consequences and their associations with caregiver status and the caregiver's

For peer review only

relationship to the patient: a survey. *BMC Cancer*, *14*(1), 541. https://doi.org/10.1186/1471-2407-14-541

29. Prostate Cancer UK. (2014). *Research into wellbeing services for men with prostate cancer-final report*. Retrieved from www.icfi.com
30. Scottish Government. (n.d.). Scotland's Census 2011. Retrieved January 6, 2023, from https://www.scotlandscensus.gov.uk/
31. Murray, E., Treweek, S., Pope, C., MacFarlane, A., Ballini, L., Dowrick, C., … May, C. (2010). Normalisation process theory: a framework for developing, evaluating and implementing complex interventions. *BMC Medicine*, *8*(1), 63. https://doi.org/10.1186/1741-7015-8-63
32. Schwarzer, R., & Luszczynska, & A. (2008). The Health Action Process Approach. *European Psychologist*, *13*(2), 141–151. https://doi.org/10.1027/1016-9040.13.2.141
33. Zhang, C. Q., Zhang, R., Schwarzer, R., & Hagger, M. S. (2019). A meta-analysis of the health action process approach. *Health psychology : official journal of the Division of Health Psychology, American Psychological Association*, *38*(7), 623–637. https://doi.org/10.1037/HEA0000728
34. Ritchie, J., & Lewis, J. (2003). *Qualitative Research Practice A Guide for Social Science Students and Researchers* (Vol. 1). London: Sage.
35. Braun, V., & Clarke, V. (Associate P. in S. S. (n.d.). *Thematic analysis : a practical guide*. Retrieved from https://books.google.com/books/about/Thematic_Analysis.html?id=eMArEAAAQBAJ
36. Scottish Government. (n.d.). Scottish Index of Multiple Deprivation. Retrieved July 5, 2019, from https://www2.gov.scot/Topics/Statistics/SIMD
37. Duncan, P., Murphy, M., Man, M.-S., Chaplin, K., Gaunt, D., & Salisbury, C. (2018). Development and validation of the Multimorbidity Treatment Burden Questionnaire (MTBQ). *BMJ open*, *8*(4), e019413. https://doi.org/10.1136/bmjopen-2017-019413
38. Gallacher, K., May, C. R., Montori, V. M., & Mair, F. S. (2011). Understanding Patients' Experiences of Treatment Burden in Chronic Heart Failure Using Normalization Process Theory. *The Annals of Family Medicine*, *9*(3), 235–243. https://doi.org/10.1370/afm.1249
39. Vijan, S., Hayward, R. A., Ronis, D. L., & Hofer, T. P. (2005). Brief report: the burden of diabetes therapy: implications for the design of effective patient-centered treatment regimens. *Journal of general internal medicine*, *20*(5), 479–82. https://doi.org/10.1111/j.1525-1497.2005.0117.x
40. Gallacher, K., Morrison, D., Jani, B., Macdonald, S., May, C. R., Montori, V. M., … Mair, F. S. (2013). Uncovering Treatment Burden as a Key Concept for Stroke Care: A Systematic Review of Qualitative Research. *PLoS Medicine*, *10*(6), e1001473. https://doi.org/10.1371/journal.pmed.1001473
41. Given, B. A., Given, C. W., Sikorskii, A., Vachon, E., & Banik, A. (2017). Medication burden of treatment using oral cancer medications. *Asia-Pacific Journal of Oncology Nursing*, *4*(4), 275–282. https://doi.org/10.4103/APJON.APJON_7_17
42. Gupta, A., Eisenhauer, E. A., & Booth, C. M. (2022). The Time Toxicity of Cancer Treatment. *Journal of Clinical Oncology*. https://doi.org/10.1200/JCO.21.02810
43. Shippee, N. D., Shah, N. D., May, C. R., Mair, F. S., & Montori, V. M. (2012). Cumulative complexity: a functional, patient-centered model of patient complexity can improve research and practice. *Journal of Clinical Epidemiology*, *65*(10), 1041–1051. https://doi.org/10.1016/j.jclinepi.2012.05.005
44. The King's Fund. (n.d.). *Long-term conditions and multi-morbidity*. Retrieved from https://www.kingsfund.org.uk/projects/time-think-differently/trends-disease-and-disability-long-term-conditions-multi-morbidity

For peer review only

45. Chao-Yueh, F., Wen-Yen, H., Chun-Shu, L., Yu-Fu, S., Cheng-Hsiang, L., Chih-Cheng, T., … Chia-Hung, K. (2017). Risk of second primary malignancies among patients with prostate cancer: A population-based cohort study. *PloS one*, *12*(4). https://doi.org/10.1371/JOURNAL.PONE.0175217
46. Coyte, A., Morrison, D. S., & McLoone, P. (2014). Second primary cancer risk - The impact of applying different definitions of multiple primaries: Results from a retrospective population-based cancer registry study. *BMC Cancer*, *14*(1), 1–11. https://doi.org/10.1186/1471-2407-14-272/TABLES/5
47. Bernhard, J., Maibach, R., Thürlimann, B., Sessa, C., & Aapro, M. S. (2002). Patients' estimation of overall treatment burden: why not ask the obvious? *Journal of clinical oncology : official journal of the American Society of Clinical Oncology*, *20*(1), 65–72. https://doi.org/10.1200/JCO.2002.20.1.65
48. Mihajlovic, M., Simic, J., Marinkovic, M., Kovacevic, V., Kocijancic, A., Mujovic, N., & Potpara, T. S. (2022). Sex-related differences in self-reported treatment burden in patients with atrial fibrillation. *Frontiers in cardiovascular medicine*, *9*. https://doi.org/10.3389/FCVM.2022.1029730
49. Sawicki, G. S., Sellers, D. E., & Robinson, W. M. (2009). High treatment burden in adults with cystic fibrosis: challenges to disease self-management. *Journal of cystic fibrosis : official journal of the European Cystic Fibrosis Society*, *8*(2), 91–96. https://doi.org/10.1016/J.JCF.2008.09.007
50. Sav, A., Salehi, A., Mair, F. S., & McMillan, S. S. (2017). Measuring the burden of treatment for chronic disease: implications of a scoping review of the literature. *BMC medical research methodology*, *17*(1), 140. https://doi.org/10.1186/s12874-017-0411-8
51. Kyei, G. (2015). A revolution in the making: a suggested model for training NHS doctors. *British Journal of General Practice*, *65*(633), 196–197. https://doi.org/10.3399/BJGP15X684505

For peer review only

Figure 1. Themes and their interactions.

For peer review only

Figure 1. Themes and their interactions.

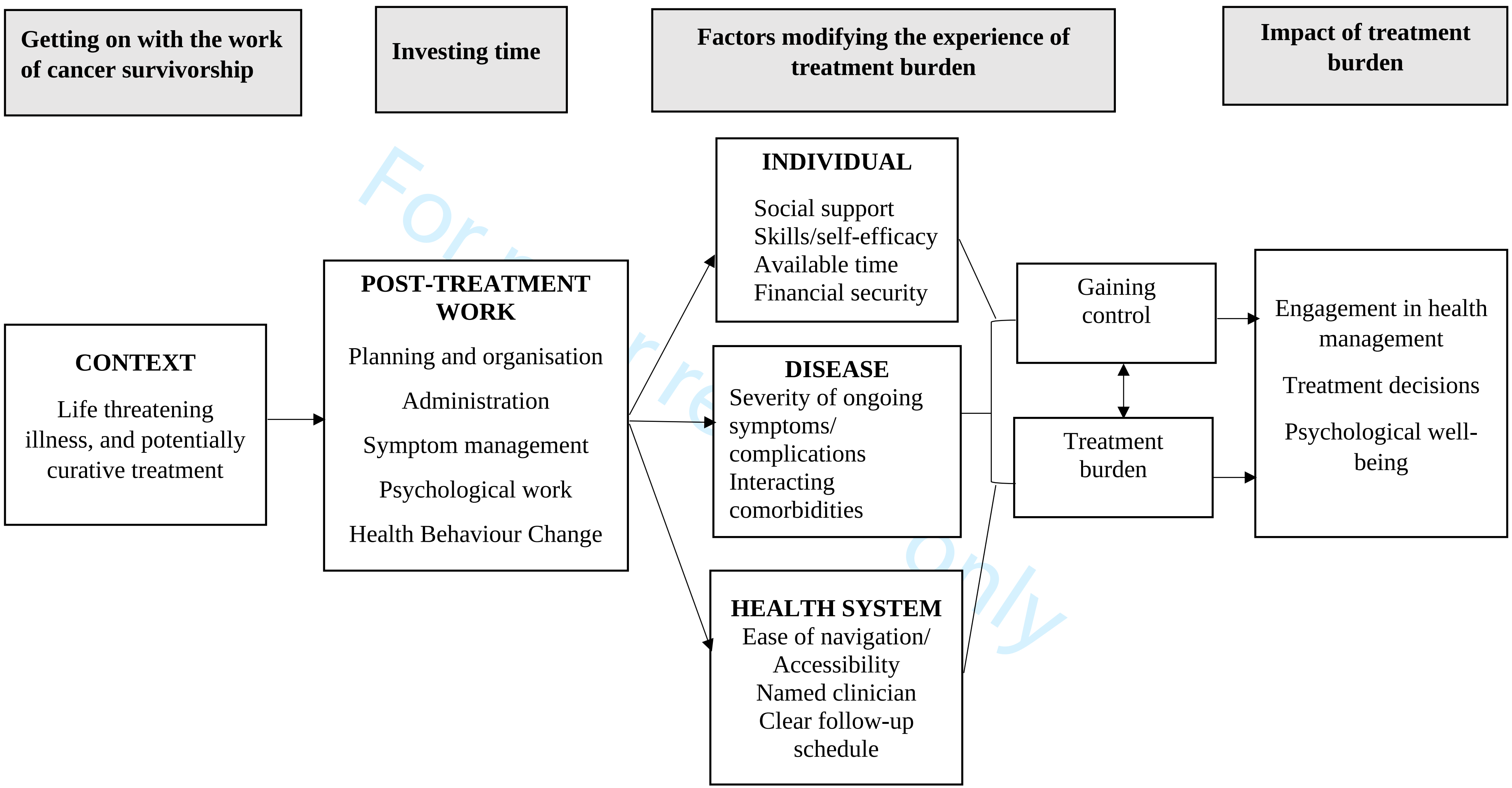

UNIVERSITY OF ABERDEEN

TOPIC GUIDE FOR PATIENT AND CAREGIVER INTERVIEWS

The Improving Cancer Aftercare Study (ICAS). Using patient and caregiver experiences to improve health services for people after prostate and colorectal cancer.

EXPLANATION

Interviews will be directed to some extent by the participants. This document sets out topics that the interviewer will aim to cover. It will be used flexibly.

INTRODUCTIONS

- Introductions and reminder that interview can be paused or stopped at any time. Audio-recording device activated.
- From previous research, we are aware that looking after yourself after cancer can involve quite a bit of work. We'd like to explore that with you.

CONTEXT

- Perhaps I can start by asking you about how your health is generally?
    - Potential prompts – comorbidities, taking multiple medications, function, employment, activity levels
- How confident do you feel about managing your health conditions? (For example, remembering to take medicines, getting help and advice when needed) Participant will be prompted to explore any difficulties

PLANNING MANAGEMENT: INITIAL DIAGNOSTIC AND TREATMENT PERIOD

- Thinking back to when you were initially diagnosed with prostate/colorectal cancer, can you tell me the story of that?

- What was important to you about the healthcare that you received then? (Prompts might include ease of access, continuity, regular appointments, information and planning, communication with health service)

- What went well?

- Were there any aspects of your care that could have been improved?

- Did you get the information that you needed? (prompts might include what kind of information was important, who was approached/available for information e.g. charities, professionals, social network)

- Can you tell me about any ways that your life changed after your diagnosis of prostate/colorectal cancer? (Prompts – more appointments, travel, new medicines, dietary changes, smoking cessation, exercise, psychological adjustment)

- I have heard it said that cancer management can be a "full time job". Do you have any thoughts about that?

For peer review only - http://bmjopen.bmj.com/site/about/guidelines.xhtml

INTERACTING WITH OTHERS

- Did anything/or anyone help you manage?
- How often were you attending hospital? (After diagnosis, during treatment, now)
- How easy or difficult is/was it for you to get help from healthcare professionals? (prompts – who was involved, specialist nurse, primary care, nursing care, has this changed over time/throughout the illness trajectory, physical access and distance to healthcare settings)
- Have family and friends been involved in any way? (Prompts might include transport, emotional support, information)
- Do you get regular tests or investigations?
- How easy/difficult do you find it to get results of your tests?

ENACTING CURRENT MANAGEMENT STRATEGIES AND GETTING FEEDBACK

Does cancer affect any aspects of a typical day or week at the moment?

  - Prompts – symptoms, self-monitoring, psychological adjustment, taking medications, strategies to remember medications

- Have you changed any of your routines or aspects of your lifestyle?
  - Prompts – diet, exercise, smoking, ***employment***, activities, ***social function***, have professionals asked the patient to make any changes?
- How confident have you felt about making these changes?
- What is the plan for your cancer follow-up? (Prompts – schedule of tests and clinic appointments, tasks that are required from patient/caregiver e.g. monitoring symptoms, taking medications, duration of follow-up, understanding of plan
- Have you had any problems scheduling or attending appointments?

SATISFACTION, SUGGESTIONS, AND SUMMING UP

- Overall, how satisfied are you with the care that you have received following your cancer diagnosis?
- Have you any suggestions for how the care could be improved?
- Some healthcare systems allow people to book or change appointments on-line, get reminders about follow-up tests, and access some results. Have you any thoughts about this?

We have covered lots of things (brief summary) – is there anything else that you would like to speak about that we haven't covered? Participant is thanked for their time.

END of INTERVIEW

For peer review only - http://bmjopen.bmj.com/site/about/guidelines.xhtml

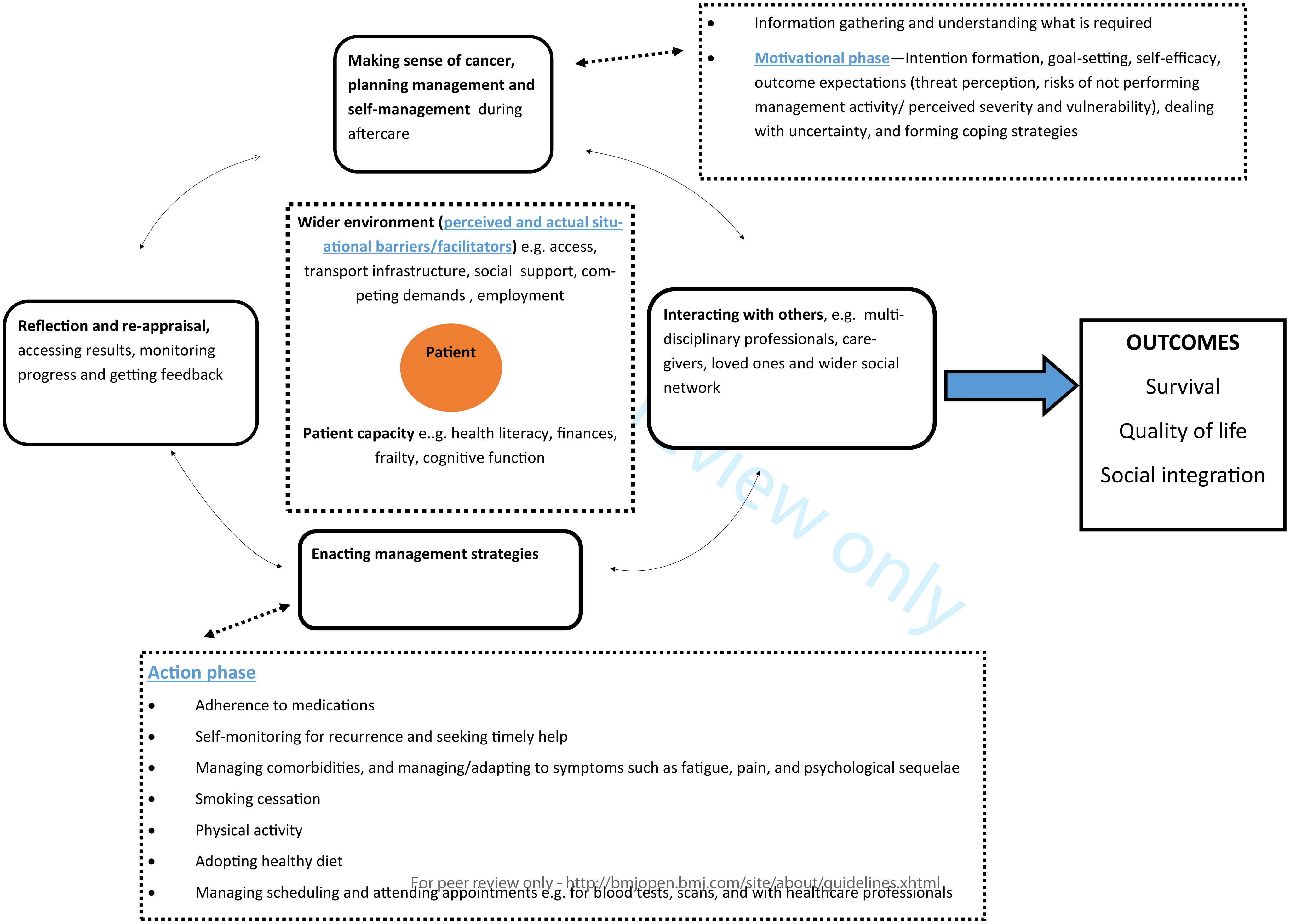

For peer review only - http://bmjopen.bmj.com/site/about/guidelines.xhtml

Supplementary Table 1. Comparisons in demographics between participants and non-participants.

	**Participants (N = 35)** **N (%)**	**Non-participants invited (N = 128)** **N (%)**	**Chi-square test of independence or Fisher Exact test *p*-value**
**Sex**			
Male	28 (80%)	105 (82%)	0.98
Female	7 (20%)	23 (18%)	
**Age**			
30-39	1 (2.9%)	0 (0%)	
40-49	0 (0%)	1 (0.8%)	
50-59	3 (8.6%)	16 (12.5%)	0.46
60-69	14 (40%)	40 (31.3%)	
70-79	14 (40%)	54 (42.2%)	
80+	3 (8.6%)	17 (13.3%)	
**Deprivation Index**			
1-2 (Highest deprivation)	2 (5.7%)	20 (15.6%)	
3-4	5 (14.3%)	34 (26.6%)	
5-6	7 (20%)	22 (17.2%)	0.06
7-8	11 (31.4%)	20 (15.6%)	
9-10 (Lowest deprivation)	9 (25.7%)	32 (25%)	
Missing	1 (2.9%)	0 (0%)	
**Urban Rural Classification**			
1 (Large urban)	17 (48.6%)	85 (66.4%)	
2 (Other urban)	1 (2.9%)	5 (3.9%)	
3 (Accessible small town)	2 (5.7%)	7 (5.5%)	0.09
4 (Remote small town)	0 (0%)	0 (0%)	
5 (Accessible rural)	10 (28.6%)	16 (12.5%)	
6 (Remote rural)	4 (11.4%)	15 (11.7%)	
Missing	1 (2.9%)	0 (0%)	
**Cancer type**			
Prostate	22 (62.9%)	80 (62.5%)	
Colorectal	13 (37.1%)	45 (35.2%)	1
Not specified	0	3 (2.3%)	

For peer review only

Treatment burden in survivors of prostate and colorectal cancer: a qualitative interview study. Standards for Reporting Qualitative Research (SRQR) Checklist

Section	Page number where relevant information can be found
**Title and abstract**	
Title: Concise description of the nature and topic of the study. Identifying the study as qualitative or indicating the approach (e.g., ethnography, grounded theory) or data collection methods (e.g., interview, focus group) is recommended.	1
Abstract: Summary of key elements of the study using the abstract format of the intended publication; typically includes background, purpose, methods, results, and conclusions.	2-3
**Introduction**	
Problem Formulation: Description and significance of the problem/phenomenon studied; review of relevant theory and empirical work; problem statement.	4-5
Purpose or research question: Purpose of the study and specific objectives or questions.	5-6
**Methods**	
Qualitative approach and research paradigm: Qualitative approach (e.g., ethnography, grounded theory, case study, phenomenology, narrative research) and guiding theory if appropriate; identifying the research paradigm (e.g., post-positivist, constructivist/interpretivist) is also recommended; rationale.	6
Researcher characteristics and reflexivity: Researchers' characteristics that may influence the research, including personal attributes, qualifications/experience, relationship with participants, assumptions, and/or presuppositions; potential or actual interaction between researchers' characteristics and the research questions, approach, methods, results and/or transferability.	8, 23
Context: Setting/site and salient contextual factors; rationale.	6, 8
Sampling strategy: How and why research participants, documents, or events were selected; criteria for deciding when no further sampling was necessary (e.g., sampling saturation); rationale.	6-7, 8
Ethical issues pertaining to human subjects: Documentation of approval by an appropriate ethics review board and participant consent, or explanation for lack thereof; other confidentiality and data security issues.	9
Data collection methods: Types of data collected; details of data collection procedures including (as appropriate) start and stop dates of data collection and analysis, iterative process, triangulation of sources/methods, and modification of procedures in response to evolving study findings; rationale.	7-8
Data collection instruments and technologies: Description of instruments (e.g., interview guides, questionnaires) and devices (e.g., audio recorders) used for data collection; if/how the instrument(s) changed over the course of the study.	7-8
Units of study: Number and relevant characteristics of participants, documents, or events included in the study; level of participation.	9-10
Data processing: Methods for processing data prior to and during analysis, including transcription, data entry, data management and security, verification of data integrity, data coding and anonymization / de-identification of excerpts.	8
Data analysis: Process by which inferences, themes, etc. were identified and developed, including the researchers involved in data analysis; usually references a specific paradigm or approach; rationale.	8
Techniques to enhance trustworthiness: Techniques to enhance trustworthiness and credibility of data analysis,(e.g., member checking, triangulation, audit trail); rationale.	8-9

For peer review only

**Results/Findings**	
Synthesis and interpretation: Main findings (e.g., interpretations, inferences, and themes); might include development of a theory or model, or integration with prior research or theory.	11-20
Links to empirical data: Evidence (e.g., quotes, field notes, text excerpts, photographs) to substantiate analytic findings.	11-20
**Discussion**	
Integration with prior work, implications, transferability, and contribution(s) to the field: Short summary of main findings, explanation of how findings and conclusions connect to, support, elaborate on, or challenge conclusions of earlier scholarship; discussion of scope of application/generalizability; identification of unique contribution(s) to scholarship in a discipline or field.	20-24
Limitations: Trustworthiness and limitations of findings	23-24
**Other**	
Conflicts of interest: Potential sources of influence or perceived influence on study conduct and conclusions; how these were managed.	25
Funding: Sources of funding and other support; role of funders in data collection, interpretation, and reporting.	25

For peer review only
